# Oldest skeleton of a plesiadapiform provides additional evidence for an exclusively arboreal radiation of stem primates in the Palaeocene

**DOI:** 10.1098/rsos.170329

**Published:** 2017-05-31

**Authors:** Stephen G. B. Chester, Thomas E. Williamson, Jonathan I. Bloch, Mary T. Silcox, Eric J. Sargis

**Affiliations:** 1 Department of Anthropology and Archaeology, Brooklyn College, City University of New York, 2900 Bedford Avenue, Brooklyn, NY 11210, USA; 2 Department of Anthropology, Graduate Center, City University of New York, 365 Fifth Avenue, New York, NY 10016, USA; 3 New York Consortium in Evolutionary Primatology, New York, NY 10024, USA; 4 New Mexico Museum of Natural History and Science, 1801 Mountain Road, NW, Albuquerque, NM 87104-1375, USA; 5 Florida Museum of Natural History, University of Florida, 1659 Museum Road, Gainesville, FL 32611-7800, USA; 6 Department of Anthropology, University of Toronto Scarborough, 1265 Military Trail, Scarborough, Ontario, CanadaM1C 1A4; 7 Department of Anthropology, Yale University, PO Box 208277, New Haven, CT 06520, USA; 8 Division of Vertebrate Paleontology, New Haven, CT 06520, USA; 9 Division of Vertebrate Zoology, Yale Peabody Museum of Natural History, New Haven, CT 06520, USA

**Keywords:** primates, plesiadapiforms, Palaeocene, paleontology, evolution

## Abstract

Palaechthonid plesiadapiforms from the Palaeocene of western North America have long been recognized as among the oldest and most primitive euarchontan mammals, a group that includes extant primates, colugos and treeshrews. Despite their relatively sparse fossil record, palaechthonids have played an important role in discussions surrounding adaptive scenarios for primate origins for nearly a half-century. Likewise, palaechthonids have been considered important for understanding relationships among plesiadapiforms, with members of the group proposed as plausible ancestors of Paromomyidae and Microsyopidae. Here, we describe a dentally associated partial skeleton of *Torrejonia wilsoni* from the early Palaeocene (approx. 62 Ma) of New Mexico, which is the oldest known plesiadapiform skeleton and the first postcranial elements recovered for a palaechthonid. Results from a cladistic analysis that includes new data from this skeleton suggest that palaechthonids are a paraphyletic group of stem primates, and that *T. wilsoni* is most closely related to paromomyids. New evidence from the appendicular skeleton of *T. wilsoni* fails to support an influential hypothesis based on inferences from craniodental morphology that palaechthonids were terrestrial. Instead, the postcranium of *T. wilsoni* indicates that it was similar to that of all other plesiadapiforms for which skeletons have been recovered in having distinct specializations consistent with arboreality.

## 1. Introduction

Plesiadapiforms are a diverse group of euarchontan mammals known from the Palaeocene and Eocene of North America, Europe and Asia. This likely paraphyletic or even polyphyletic group of mammals [1,2] has long been considered primate-like based on aspects of their dental morphology, and the most recent phylogenetic analyses suggest that they are either stem primates [3–6] or stem members of Primatomorpha (Primates + Dermoptera) [7,8]. The fossil record suggests that primates and other placental mammals diversified following the Cretaceous–Paleogene boundary [9]. The diversification of primates (*sensu lato*) was not instantaneous, as fossil-bearing strata from the Puercan North American Land Mammal ‘Age’ (NALMA) have only produced a small number of primitive plesiadapiform species (e.g. [10–12]), and the first occurrences of a wider diversity of plesiadapiform families (Palaechthonidae, Paromomyidae, Picrodontidae, Carpolestidae and Plesiadapidae) are not documented until the following Torrejonian NALMA [1,2].

Although fragmentary, the dentitions of plesiadapiforms from the Torrejonian NALMA represent a variety of tooth forms that likely reflect a diversity of ecological specializations [13]. Postcranial fossils of early and middle Palaeocene plesiadapiforms (e.g. Palaechthonidae and Picrodontidae) are even more rare, so locomotor habits of virtually all of these taxa that predate the late Palaeocene are unknown. Alternatively, dentally associated partial skeletons from the late Palaeocene and early Eocene representing four plesiadapiform families (Plesiadapidae, Carpolestidae, Paromomyidae and Micromomyidae) are known, and indicate that members of those families had a variety of positional behaviours, yet were all clearly arboreal [14]. More specifically, they likely frequently used orthograde (upright) postures while clinging and climbing on vertical supports and were capable of grasping small diameter branches with their hands and feet (e.g. [3,15–17]). However, these skeletons almost all represent relatively late occurring taxa that are well nested within their respective clades [14]. With the exception of several specimens of plesiadapids (e.g. [18]) and isolated tarsals attributed to *Purgatorius* [6], postcrania of early and middle Palaeocene plesiadapiforms, such as palaechthonids, have been lacking.

**Figure 1.**
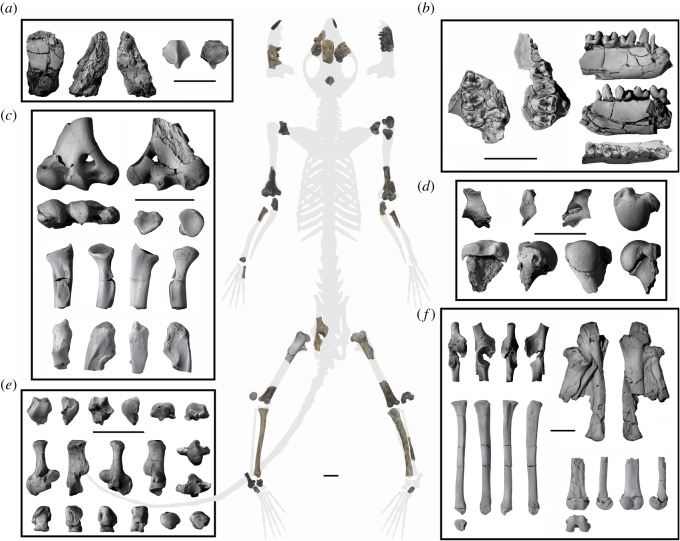
Skeleton composite of *Torrejonia wilsoni* (NMMNH P-54500). Most elements of the composite skeleton are in ventral view, but some elements are oriented differently to better illustrate articular surfaces. Descriptions and orientations of skeletal elements organized from left to right and then from top to bottom: (*a*) cranial fragment of R frontal in dorsal, left lateral, right lateral views; cranial fragment of parietals in dorsal, ventral views. (*b*) R maxilla M1–M3 in occlusal view; L maxilla P4, M2–M3 in occlusal view; R dentary p2–m2 in buccal, lingual, occlusal views (also see [13]). (*c*) R distal humerus in ventral, dorsal, distal views; R distal radial epiphysis in distal view; R proximal radius in proximal, ventral, lateral, dorsal, medial views; R proximal ulna in ventral, lateral, dorsal, medial views. (*d*) R scapula fragment in ventral, lateral, dorsal views; L proximal humerus in proximal, ventral, lateral, dorsal, medial views. (*e*) R partial astragalus in dorsal, lateral, plantar, medial, proximal, distal views; R calcaneus in dorsal, lateral, plantar, medial, proximal, distal views; R cuboid in dorsal, lateral, plantar, medial, proximal, distal views. (*f*) R partial innominate in ventral, lateral, dorsal, medial views; concretion with proximal femora and L tibia with L proximal femur in ventral view, R proximal femur in ventral view; R tibia in ventral, lateral, dorsal, medial, distal views; L distal femur in ventral, lateral, dorsal, medial, distal views. Scale bars, 1 cm.

The early occurrence and hypothesized relatively basal position of the Palaechthonidae among plesiadapiforms suggests this family could be important for understanding euarchontan and early primate evolution, with some palaechthonids proposed to be ancestors of microsyopids [19] or paromomyids [2]. Based on the only previously reported non-dental specimen of a palaechthonid, a partial cranium of *Plesiolestes nacimienti*, aspects of craniodental morphology (e.g. the presence of a large infraorbital foramen (IOF)) were cited as evidence that *P. nacimienti* was predominantly terrestrial, with adaptations similar to those of a hedgehog [20,21]. This inference was further extended to primitive primates more generally, implying that arboreality evolved later in primate evolution [21]. This hypothesis is assessed here by analysing the first postcrania known for the Palaechthonidae, a new partial skeleton of *Torrejonia wilsoni* (NMMNH P-54500), from the late Torrejonian (To3) of the Nacimiento Formation, San Juan Basin, New Mexico (figure 1).

This partial skeleton was found at locality NMMNH L-6898 in the Nacimiento Formation, San Juan Basin, New Mexico (collected under Bureau of Land Management excavation permit NM07-002E to T.E.W.). This locality is within the Torrejonian (To3) *Mixodectes pungens* zone of Torrejon Wash [22], which yields the type fauna for the Torrejonian NALMA [23,24]. The site is located within a normal polarity zone correlated with Chron C27n, which constrains the age of the site to about 62 Ma [25–27]. What is interpreted to be a partial skeleton of a single individual of *T. wilsoni* was recovered mixed with partial skeletons of two other mammals (see the electronic supplementary material), including a nearly complete skeleton of *M. pungens* (NMMNH P-54501) and a less complete skeleton of the eutherian mammal *Acmeodon secans* (Cimolestidae; NMMNH P-54499).

Postcrania of *T. wilsoni* were readily distinguished from those of *M. pungens* by their smaller size. *Torrejonia wilsoni* is similar in size to *A. secans*, but the morphology of many of the bones attributed to *T. wilsoni* very closely resembles that of other dentally associated plesiadapiform skeletons. Also, although the skeleton of *Torrejonia* has a fully erupted adult dentition, many of the long bone epiphyses remain unfused, a pattern that has been previously documented for other plesiadapiforms (e.g. [28]). By contrast, epiphyseal fusion of postcranial elements attributed to *A. secans* indicates that this was a relatively more mature individual. Many additional elements such as fragmentary vertebrae, metapodials and phalanges are more difficult to distinguish between these two mammals, so they are not considered further here. There are no duplicated tooth positions or skeletal elements for *T. wilsoni*, which suggests that only one individual of this species was present.

## 2. Description and functional assessment of postcranium

NMMNH P-54500 includes left i1, right dentary with p2–m2 and alveoli for i1, i2, c1; left dentary with m2, m3 talonid; left maxilla with P4, M2–M3 and roots for P2, P3, M1; right isolated I1, right maxilla with M1–M3 (see descriptions of teeth in [13]), two associated cranial fragments and many appendicular skeletal elements (figure 1). The larger cranial fragment preserves a portion of the right frontal with a rostral margin that likely represents a broad sutural contact with the right nasal. The lateral aspect of the frontal preserves a portion of the temporal crest and there is no evidence of a postorbital process, but it is unclear whether the orbital margin is preserved far enough caudally to fully assess this. The caudal aspect of the skull roof is missing, which exposes what might be the natural endocast of the rostral portion of the right cerebrum. The smaller cranial fragment represents portions of both parietals, fused together, with a narrow sagittal crest on the dorsal surface. The ventral surface preserves depressions on either side of the midline for what were likely the caudal colliculi.

The glenohumeral joint of *T. wilsoni* is known from the lateral portions of both scapulae and the left proximal humerus (figure 1), which provide evidence for a mobile shoulder as in arboreal mammals. The glenoid fossa is concave and pear-shaped (i.e. wider inferiorly) and smaller than the dimensions of the convex humeral head. The humeral head extends superiorly beyond the well-developed greater and lesser tuberosities, which allows great mobility at the shoulder joint [29]. The lesser tuberosity is large and projects medially as in other arboreal euarchontans, and would have provided a large area of insertion for the subscapularis muscle, which medially rotates the humerus during vertical climbing [16,29].

**Figure 2.**
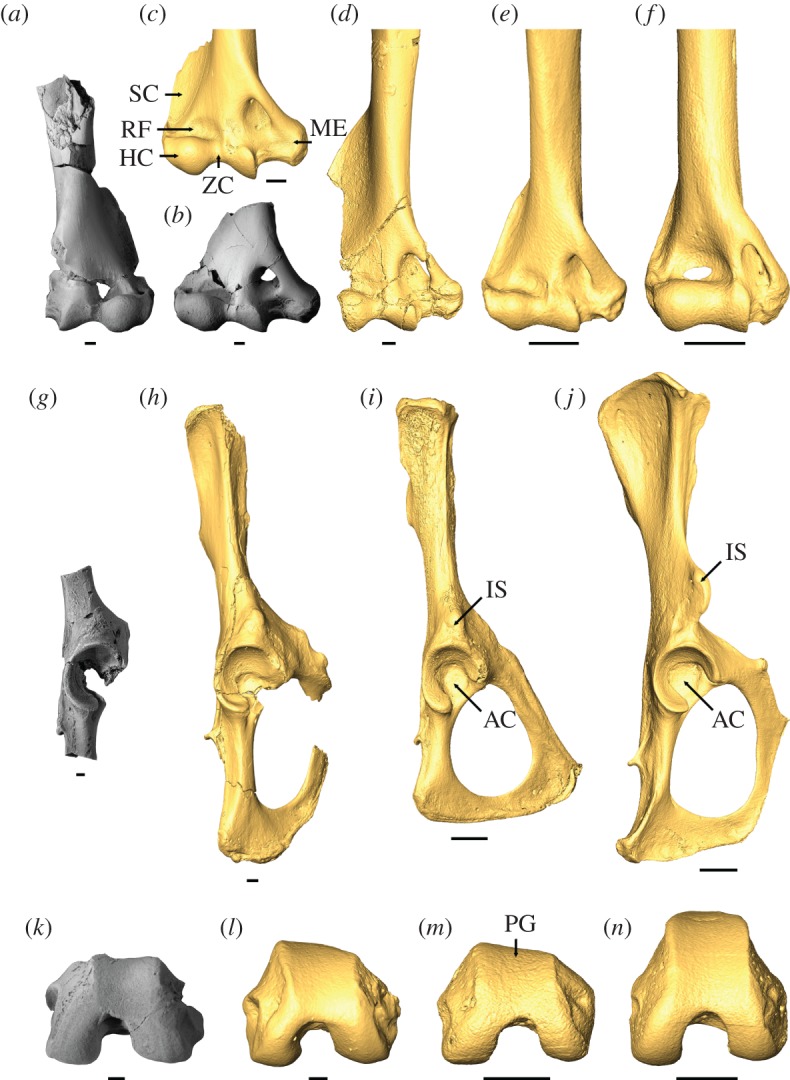
Comparison of distal humerus, innominate and distal femur of *Torrejonia wilsoni* (NMMNH P-54500) with those of other euarchontan mammals. Photographs of (*a*) left and (*b*) right distal humeri of *T. wilsoni* and micro X-ray CT scan reconstructions of those of paromomyid plesiadapiforms (*c*) cf. *Phenacolemur simonsi* (USNM 442260) and (*d*) *Ignacius clarkforkensis* (UM 108210), and extant treeshrews (*e*) arboreal *Ptilocercus lowii* (MCZ 51736) and (*f*) terrestrial *Tupaia gracilis* (FMNH 140928) in ventral view. Photograph of (*g*) partial right innominate of *T. wilsoni* and micro X-ray CT scan reconstructions of innominates of (*h*) *I. clarkforkensis* (UM 82606), (*i*) arboreal *P. lowii* (MCZ 51736) and (*j*) terrestrial *T. gracilis* (FMNH 140928) in lateral view. Photograph of (*k*) left distal femur of *T. wilsoni* and micro X-ray CT scan reconstructions of distal femora of (*l*) *I. clarkforkensis* (UM 82606), (*m*) arboreal *P. lowii* (MCZ 51736) and (*n*) terrestrial *T. gracilis* (FMNH 140928) in distal view. Some elements reversed for clarity. Scale bars, 1 mm. AC, acetabulum; HC, humeral capitulum; IS, anterior inferior iliac spine; ME, medial epicondyle; PG, patellar groove; RF, radial fossa; SC, supinator crest; ZC, zona conoidea. See the electronic supplementary material for institutional abbreviations.

The elbow joint of *T. wilsoni* is known from distal humeri, proximal ulnae and proximal radii (figures 1 and 2). They provide evidence for a mobile elbow joint, habitual flexion of the forearm, and capability for manual grasping, as required in arboreal locomotion and vertical positional behaviours. The prominent, laterally flaring supinator crest on the left humerus is the origin for brachioradialis, which would have contributed to flexion of the forearm. Both humeri preserve deep radial fossae and shallow olecranon fossae, allowing for complete flexion but limited extension of the antebrachium, respectively. A large and proximally positioned bicipital tuberosity on both radii suggests a large biceps brachii, a forearm flexor and supinator that is important for climbing in arboreal locomotion [29,30]. The globular humeral capitulum and round, excavated radial central fossa would have enabled a great degree of radial rotation, allowing extensive capability for supination and pronation of the forearm and hand [31]. The zona conoidea separates the capitulum from the trochlea, allowing freer rotation of the radius in relation to the ulna [32]. The medial epicondyle is very wide and provides a large area of origin for the wrist and digital flexors [29,30]. These muscles are important for flexion of the digits during manual grasping, and would be beneficial for an arboreal mammal exhibiting positional behaviours on the branches of trees.

The hip joint of *T. wilsoni* is known from proximal femora and a partial right innominate (figures 1 and 2). They provide evidence for a mobile hip and flexed thigh. The acetabulum is craniocaudally elliptical, allowing a high degree of mobility at the hip joint for great ranges of abduction and lateral rotation, as in other arboreal euarchontans, and in contrast to a more circular acetabulum that restricts mobility in terrestrial taxa [33]. The acetabulum is also cranially buttressed with an expanded articular surface that likely reflects loads incurred during orthograde postures on vertical supports [16,29]. The femur has a large lesser trochanter for insertion of iliopsoas, a major hip flexor and a small third trochanter for insertion of gluteus superficialis, which suggests that *Torrejonia* was not powerfully extending its thigh and had a more habitually flexed hind limb [33].

Aspects of the knee joint of *Torrejonia* can be assessed from the innominate, left distal femur and proximal tibiae (figures 1 and 2), and provide evidence for a habitually flexed knee with no evidence for specialized leaping or terrestrial running. The innominate has a small anterior inferior iliac spine providing a small area of origin for rectus femoris, which likely reflects that *Torrejonia* had a habitually flexed knee and was not powerfully extending its leg. By contrast, terrestrial euarchontans such as some treeshrews have a larger anterior inferior iliac spine for powerful leg extension when running on the ground [33]. The distal femora are fairly shallow dorsoventrally with a shallow and proximally restricted patellar groove, and although the proximal tibiae are missing their proximal epiphyses they appear to have lacked a large tibial crest or tuberosity. These features also suggest a more habitually flexed knee posture in contrast to leaping or terrestrial running specialists, which have a deeper knee for increasing leverage of quadriceps femoris for powerful extension of the leg [33].

**Figure 3.**
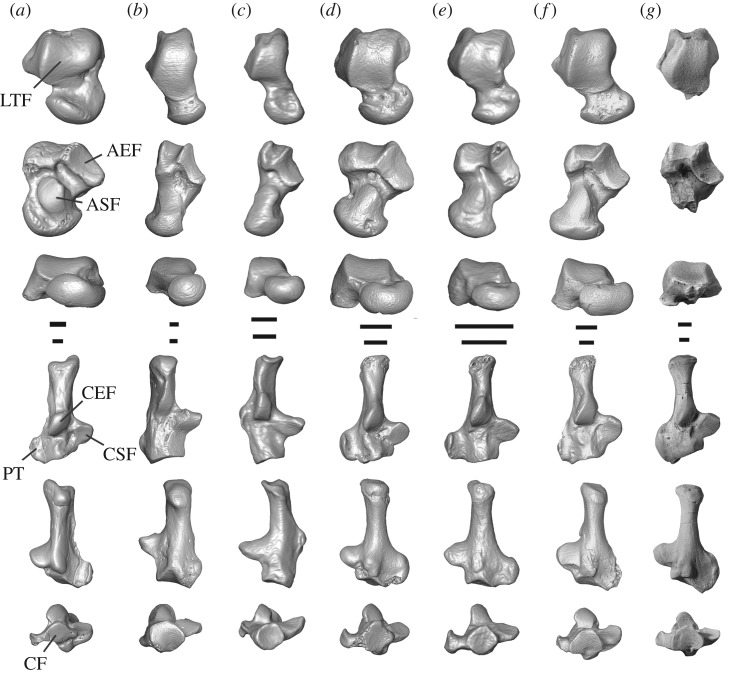
Comparison of astragalus and calcaneus of *Torrejonia wilsoni* (NMMNH P-54500) with those of other euarchontan mammals. Columns illustrate micro X-ray CT scan reconstructions of tarsals of (*a*) terrestrial condylarth cf. *Protungulatum* (AMNH 118260, 118060), (*b*) colugo *Cynocephalus* (UNSM 15502, AMNH 207001), (*c*) arboreal treeshrew *Ptilocercus* (USNM 488072), (*d*) purgatoriid plesiadapiform cf. *Purgatorius* (UCMP 197507, 197517), (*e*) micromomyid plesiadapiform *Dryomomys* (UM 41870), (*f*) paromomyid plesiadapiform *Ignacius* (USNM 442235, 442240), and photographs of (*g*) *Torrejonia wilsoni*. Right astragali (rows 1–3) and calcanei (rows 4–6) in dorsal (top), plantar (middle), distal (bottom) views. Some elements reversed for clarity. Scale bars, 1 mm. AEF, astragalar ectal facet; ASF, astragalar sustentacular facet; CEF, calcaneal ectal facet; CF, calcaneal cuboid facet; CSF, calcaneal sustentacular facet; LTF, astragalar lateral tibial facet; PT, calcaneal peroneal tubercle. See the electronic supplementary material for institutional abbreviations.

The right distal tibia, astragalus, calcaneus and cuboids of *Torrejonia* (figures 1 and 3) provide evidence for mobile ankle joints allowing increased inversion and eversion to adjust to uneven and variable arboreal supports. The upper ankle joint is represented by a distal tibia with a short medial malleolus and a dorsolaterally oriented and ungrooved articular surface that mirrors the lateral tibial facet of the astragalus. The extension of the astragalar lateral tibial facet onto the astragalar neck suggests a habitually dorsiflexed foot in *Torrejonia*, as is typical of mammals that cling to vertical supports such as tree trunks [34]. The calcaneal ectal facet is longer than the corresponding astragalar ectal facet allowing translation, and the calcaneal sustentacular facet is continuous distally onto the body, allowing increased inversion and eversion at the lower ankle joint [6,34]. The calcaneus is not distally elongated, in contrast to the condition in leaping euprimates. The peroneal process is very large, projects distolaterally, and has a groove on the lateral side for the tendon of peroneus longus, a muscle that would have contributed to eversion of the foot. The calcaneal cuboid facet is concave with a well-developed plantar pit, mirroring the proximal articular surface of the cuboids. Both features would have contributed to high degrees of inversion and eversion at the transverse tarsal joint.

## 3. Phylogenetic analysis

The new skeleton of *T. wilsoni* was coded into a morphological data matrix [4] designed to assess relationships within Euarchontoglires, which includes multiple outgroups, extant and fossil members of that clade [4–6]. This character matrix [4] was revised by adding two taxa, palaechthonids *T. wilsoni* and *Plesiolestes nacimienti* (the only other palaechthonid known from more than dental fossils), and excluding apatemyids (see the electronic supplementary material). Maximum-parsimony analysis was conducted using TNT (v. 1.5) software subsidized by the Willi Hennig Society [35]. All multistate characters were treated as unordered. New Technology Search was implemented using 100 replications as the starting point for each hit, 200 parsimony ratchet iterations, 10 rounds of tree drifting, 10 rounds of tree fusing and sectorial searching. The resulting most parsimonious tree (MPT) was used as a starting tree in a Traditional Heuristic Search that was carried out using tree-bisection and reconnection. Absolute Bremer Support values were calculated using the suboptimal trees found in the search performed using the bremer.run script [36], which rigorously searches incrementally for suboptimal trees, with up to 10 000 trees held, increasing the score by 1 up to a maximum of 10 steps longer than the MPTs.

**Figure 4.**
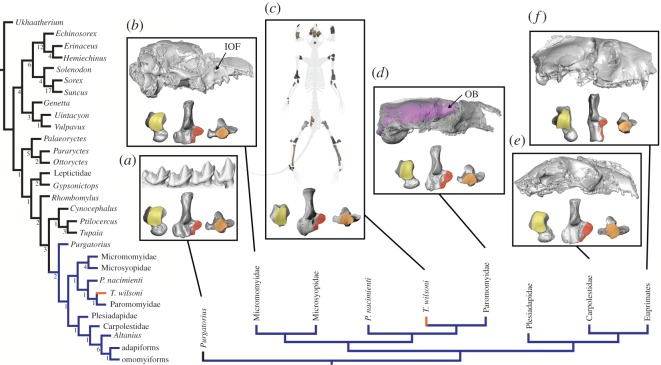
Hypothesis of evolutionary relationships of *Torrejonia wilsoni* and other eutherian mammals. (Left) Resulting single most parsimonious cladogram based on modified morphological dataset of Bloch *et al.* [4], sampling a total of 240 morphological characters (68 postcranial, 45 cranial and 127 dental) with Primates *sensu lato* indicated in blue and *Torrejonia wilsoni* supported as a stem primate and indicated in orange. Numbers below branches represent Absolute Bremer Support values. See the electronic supplementary material for detailed methods, descriptions of morphological characters, specimens examined (also see [5]), and the taxon-character matrix in TNT format. (Bottom) Simplified subset of resulting tree topology focused on Primates. Boxes (*a*–*f*) illustrate tarsals of select primates with great mobility at the upper ankle joint (yellow: lateral tibial facet extends distally onto neck of astragalus in dorsal view), lower ankle joint (red: sustentacular facet extends distally onto body of calcaneus in dorsal view) and transverse tarsal joint (orange: round, concave cuboid facet of calcaneus in distal view) indicating arboreality. Boxes (*a*–*f*) also illustrate micro X-ray CT scan reconstructions of (*a*) purgatoriid *Purgatorius unio* p4-m3 (UCMP 107406) with tall molar cusps in buccal view, (*b*) micromomyid *Dryomomys szalayi* cranium (UM 41870) in right lateral view with large IOF, (*c*) *Torrejonia wilsoni* partial skeleton (NMMNH P-54500), (*d*) paromomyid *Ignacius graybullianus* cranium (USNM 421608) in right lateral view with relatively large olfactory bulbs (OB) of endocast (violet), (*e*) carpolestid *Carpolestes simpsoni* cranium (USNM 482354) in right lateral view and tarsals (UM 101963) and (*f*) notharctid *Notharctus tenebrosus* cranium (AMNH 127167) in right lateral view. Some elements reversed for clarity. See figure 3 legend for specimen numbers of tarsals not listed above. See the electronic supplementary material for institutional abbreviations.

The cladistic analysis yielded a single MPT. This tree supports Palaechthonidae as a paraphyletic group of stem primates with *T. wilsoni* more closely related to paromomyids than to *P. nacimienti*, though both palaechthonids form a clade with Paromomyidae (figure 4). One additional step (998 total steps) is required to recover a monophyletic Palaechthonidae (i.e. *P. nacimienti* and *T. wilsoni* as sister taxa), which is the sister taxon to Paromomyidae. Ten additional steps (1007 total steps) are required to recover a clade consisting of *P. nacimienti*, *T. wilsoni* and Microsyopidae, so the hypothesis that palaechthonids and microsyopids are closely related is not as well supported in this analysis. Broader results of this analysis include all plesiadapiforms as stem primates, and Primates (*sensu lato*) as the sister taxon to Sundatheria (Dermoptera + Scandentia).

Though there is an empirical basis for accurate primate phylogeny reconstruction based on morphological data alone [37], our analysis did not recover Primatomorpha (Dermoptera + Primates), which was most recently supported by phylogenomic data [38]. Therefore, an additional maximum-parsimony analysis was performed in which Primatomorpha (e.g. [38]) was constrained with a scaffold that forced treeshrews (*Ptilocercus* and *Tupaia*) outside a Primatomorpha clade consisting of a colugo and unambiguous fossil primates (*Cynocephalus* + adapiforms + omomyiforms). Results are somewhat less resolved within Primates, but all plesiadapiforms are still supported as stem primates and the relationships of *T. wilsoni*, *P. nacimienti* and paromomyids are unchanged (see the electronic supplementary material).

## 4. Discussion and conclusion

Based on craniodental morphology, the palaechthonid *P. nacimienti* was reconstructed as a predominantly terrestrial early primate that relied heavily on tactile and olfactory information like a hedgehog or some other ground-dwelling insectivore [21]. Dental features used to support this hypothesis include the relatively tall lower molar trigonid cusps of *P. nacimienti*, which closely resemble those of the oldest known plesiadapiform, *Purgatorius*, and suggest an omnivorous diet that included a large proportion of insects [20,21]. Cranial features used to support this hypothesis include small, laterally oriented and widely separated orbits, and a large IOF that suggests the presence of many vibrissae and a well-innervated snout [21]. It was further proposed that primitive insectivorous and terrestrial primates such as *P. nacimienti* were similar to the last common ancestor of plesiadapiforms and euprimates, and that arboreality evolved in parallel in these two groups as they shifted to a more plant-based diet [21].

The greatly improved plesiadapiform fossil record demonstrates that some of the cranial features previously cited as informative for a terrestrial substrate preference in *P*. *nacimienti*, such as relatively small and laterally oriented orbits and large infraorbital foramina, occur in other plesiadapiforms, such as the micromomyid *Dryomomys*, that have postcranial features indicative of arboreality (figure 4). Furthermore, recent studies of endocranial anatomy provide additional evidence for limited visual processing and strong olfaction in arboreal plesiadapiforms (e.g. paromomyid *Ignacius* [39]). Also, isolated tarsal bones have recently been attributed to the geologically oldest plesiadapiform *Purgatorius*, and they suggest that fairly insectivorous primitive plesiadapiforms were arboreal [6]. These discoveries demonstrate that the suggested links between terrestriality and insectivorous dental traits, as well as terrestriality and cranial traits associated with less specialized vision, are not upheld in the early primate fossil record (figure 4).

Analysis of the partial skeleton of *T. wilsoni* demonstrates that this first palaechthonid known from postcrania was arboreal and had capabilities for clinging and climbing on vertical supports like other plesiadapiforms. Although arboreality in *T. wilsoni* is certainly not direct evidence for arboreality in *P. nacimienti*, the latter may have been arboreal given that it is phylogenetically bracketed by arboreal taxa such as *T. wilsoni*, paromomyids and micromomyids (figure 4) [3,14]. If it is discovered that *P. nacimienti* or any other taxon nested within Primates has features of the postcranium related to terrestriality, they would be secondarily derived. This implies that arboreality did not evolve in parallel in separate groups of primitive primates, but rather that primates are primitively arboreal (e.g. [3,6,15,34]) and plesiadapiforms represent an exclusively arboreal radiation based on all the relevant skeletal evidence, including the new partial skeleton of *T. wilsoni* analysed here.

## Supplementary Material

Electronic Supplementary Material: Oldest skeleton of a plesiadapiform provides evidence for an exclusively arboreal radiation of stem primates in the Paleocene

## Data accessibility

All supporting data are available in the electronic supplementary material.

## References

[1] Szalay FS, Delson E 1979 Evolutionary history of the primates, p. 580 New York, NY: Academic Press.

[2] Silcox MT, Bloch JI, Boyer DM, Chester SGB, López-Torres S 2017 The evolutionary radiation of plesiadapiforms. Evol. Anthropol. 26, 74–94. (doi:10.1002/evan.21526) 28429568 10.1002/evan.21526

[3] Bloch JI, Silcox MT, Boyer DM, Sargis EJ 2007 New Paleocene skeletons and the relationship of plesiadapiforms to crown-clade primates. Proc. Natl Acad. Sci. USA 104, 1159–1164. (doi:10.1073/pnas.0610579104) 17229835 10.1073/pnas.0610579104 PMC1783133

[4] Bloch JI, Chester SGB, Silcox MT 2016 Cranial morphology of Paleocene micromomyid plesiadapiform *Dryomomys szalayi* (Mammalia, Primates): implications for early primate evolution. J. Hum. Evol. 96, 58–81. (doi:10.1016/j.jhevol.2016.04.001) 27343772 10.1016/j.jhevol.2016.04.001

[5] Silcox MT, Bloch JI, Boyer DM, Houde P 2010 Cranial anatomy of Paleocene and Eocene *Labidolemur kayi* (Mammalia: Apatotheria), and the relationships of the Apatemyidae to other mammals. Zool. J. Linn. Soc. 160, 773–825. (doi:10.1111/j.1096-3642.2009.00614.x)

[6] Chester SGB, Bloch JI, Boyer DM, Clemens WA 2015 Oldest known euarchontan postcrania and affinities of Paleocene *Purgatorius* to Primates. Proc. Natl Acad. Sci. USA 112, 1487–1492. (doi:10.1073/pnas.1421707112) 25605875 10.1073/pnas.1421707112 PMC4321231

[7] Ni X et al. 2013 The oldest known primate skeleton and early haplorhine evolution. Nature 498, 60–64. (doi:10.1038/nature12200) 23739424 10.1038/nature12200

[8] Ni X, Li Q, Li K, Beard KC 2016 Oligocene primates from China reveal divergence between African and Asian primate evolution. Science 352, 673–677. (doi:10.1126/science.aaf2107) 27151861 10.1126/science.aaf2107

[9] O'Leary MA et al. 2013 The placental mammal ancestor and the post-K-Pg radiation of placentals. Science 339, 662–667. (doi:10.1126/science.1229237) 23393258 10.1126/science.1229237

[10] Van Valen L, Sloan RE 1965 The earliest primates. Science 150, 743–745. (doi:10.1126/science.150.3697.743) 5891702 10.1126/science.150.3697.743

[11] Van Valen LM 1994 The origin of the plesiadapid Primates and the nature of *Purgatorius*. Evol. Monographs 15, 1–79.

[12] Fox RC, Scott CS, Buckley GA 2015 A ‘giant'purgatoriid (Plesiadapiformes) from the Paleocene of Montana, USA: mosaic evolution in the earliest primates. Palaeontology 58, 277–291. (doi:10.1111/pala.12141)

[13] Silcox MT, Williamson TE 2012 New discoveries of early Paleocene (Torrejonian) primates from the Nacimiento Formation, San Juan Basin, New Mexico. J. Hum. Evol. 63, 805–833. (doi:10.1016/j.jhevol.2012.09.002) 23084622 10.1016/j.jhevol.2012.09.002

[14] Bloch JI, Boyer DM 2007 New skeletons of Paleocene-Eocene Plesiadapiformes: a diversity of arboreal positional behaviors in early primates. In Primate origins: adaptations and evolution (eds Ravosa MJ, Dagosto M), pp. 535–582. New York, NY: Plenum Press.

[15] Szalay FS, Tattersall I, Decker RL 1975 Phylogenetic relationships of *Plesiadapis* - postcranial evidence. In Approaches to primate paleobiology (ed. Szalay FS), pp. 136–166. Basel, Switzerland: Karger. 1112090

[16] Beard KC 1991 Vertical postures and climbing in the morphotype of Primatomorpha: implications for locomotor evidence in primate history. In Origines de La Bipédie Chez les Hominidés (eds Coppens Y, Senut B), pp. 79–87. Paris: Editions du CNRS Cahiers de Paleoanthropologie.

[17] Boyer DM, Yapuncich GS, Chester SGB, Bloch JI, Godinot M 2013 Hands of early primates. Yearb. Phys. Anthropol. 57, 33–78. (doi:10.1002/ajpa.22392) 10.1002/ajpa.22392 24249591

[18] Simpson GG 1935 The Tiffany fauna, upper Paleocene. II. Structure and relationships of *Plesiadapis*. Am. Mus. Novit. 816, 1–30.

[19] Bown TM, Gingerich PD 1973 The Paleocene primate *Plesiolestes* and the origin of Microsyopidae. Folia Primatol. 19, 1–8. (doi:10.1159/000155511) 4718725 10.1159/000155511

[20] Kay RF, Cartmill M 1974 Skull of *Palaechthon nacimienti*. Nature 252, 37–38. (doi:10.1038/252037a0)

[21] Kay RF, Cartmill M 1977 Cranial morphology and adaptations of *Palaechthon nacimienti* and other Paromomyidae (Plesiadapoidea, ?Primates), with a description of a new genus and species. J. Hum. Evol. 6, 19–53. (doi:10.1016/S0047-2484(77)80040-7)

[22] Williamson TE 1996 The beginning of the age of mammals in the San Juan Basin, New Mexico; biostratigraphy and evolution of Paleocene mammals of the Nacimiento Formation. New Mexico Mus. Nat. Hist. Sci. Bull. 8, 1–141.

[23] Wood HE, Chaney RW, Clark JM, Colbert EH, Jepsen GL, Reeside JR Jr, Stock C 1941 Nomenclature and correlation of the North American continental Tertiary. Geol. Soc. Am. Bull. 52, 1–48. (doi:10.1130/GSAB-52-1)

[24] Lofgren DL, Lillegraven JA, Clemens WA, Gingerich PD, Williamson TE 2004 Paleocene biochronology: the Puercan through Clarkforkian land mammal ages. In Late Cretaceous and Cenozoic mammals of North America (ed. Woodburne MO), pp. 43–105. New York, NY: Columbia University Press.

[25] Kuiper KF, Deino A, Hilgen FJ, Krijgsman W, Renne PR, Wijbrans JR 2008 Synchronizing rock clocks of Earth history. Science 320, 500–504. (doi:10.1126/science.1154339) 18436783 10.1126/science.1154339

[26] Luterbacher HP et al. 2004 The Paleogene period. In A geologic time scale 2004 (eds Gradstein FM, Ogg JG, Smith AG), pp. 384–408. Cambridge, UK: Cambridge University Press.

[27] Ogg JG 2012 Geomagnetic polarity time scale. In The geologic time scale 2012 (eds Gradstein FM, Ogg JG, Schmitz MD, Ogg G), pp. 85–113. Oxford, UK: Elsevier.

[28] Chester SGB, Bloch JI 2013 Systematics of Paleogene Micromomyidae (Euarchonta, Primates) from North America. J. Hum. Evol. 65, 109–142. (doi:10.1016/j.jhevol.2013.04.006) 23850536 10.1016/j.jhevol.2013.04.006

[29] Sargis EJ 2002 Functional morphology of the forelimb of tupaiids (Mammalia, Scandentia) and its phylogenetic implications. J. Morphol. 253, 10–42. (doi:10.1002/jmor.1110) 11981802 10.1002/jmor.1110

[30] Argot C 2001 Functional-adaptive anatomy of the forelimb in the Didelphidae, and the paleobiology of the Paleocene marsupials *Mayulestes ferox* and *Pucadelphys andinus*. J. Morphol. 247, 51–79. (doi:10.1002/1097-4687(200101)247:1<51::AID-JMOR1003>3.0.CO;2-#) 11124686 10.1002/1097-4687(200101)247:1<51::AID-JMOR1003>3.0.CO;2-#

[31] Szalay FS, Dagosto M 1980 Locomotor adaptations as reflected on the humerus of Paleogene primates. Folia Primatol. 34, 1–45. (doi:10.1159/000155946) 7002751 10.1159/000155946

[32] Gebo DL, Sargis EJ 1994 Terrestrial adaptations in the postcranial skeletons of guenons. Am. J. Phys. Anthropol. 93, 341–371. (doi:10.1002/ajpa.1330930306) 8042696 10.1002/ajpa.1330930306

[33] Sargis EJ 2002 Functional morphology of the hindlimb of tupaiids (Mammalia, Scandentia) and its phylogenetic implications. J. Morphol. 254, 149–185. (doi:10.1002/jmor.10025) 12353299 10.1002/jmor.10025

[34] Szalay FS, Drawhorn G 1980 Evolution and diversification of the Archonta in an arboreal milieu. In Comparative biology and evolutionary relationships of tree shrews (ed. Luckett WP), pp. 133–169. New York, NY: Plenum Press.

[35] Goloboff PA, Catalano SA 2016 TNT version 1.5, including a full implementation of phylogenetic morphometrics. Cladistics 32, 221–238. (doi:10.1111/cla.12160) 10.1111/cla.12160 34727670

[36] Goloboff PA, Farris JS, Nixon KC 2008 TNT, a free program for phylogenetic analysis. Cladistics 24, 774–786. (doi:10.1111/j.1096-0031.2008.00217.x)

[37] Pattinson DJ, Thompson RS, Piotrowski AK, Asher RJ 2015 Phylogeny, paleontology, and primates: do incomplete fossils bias the tree of life? Syst. Biol. 64, 169–186. (doi:10.1093/sysbio/syu077) 25239212 10.1093/sysbio/syu077

[38] Mason VC et al. 2016 Genomic analysis reveals hidden biodiversity within colugos, the sister group to primates. Sci. Adv. 2, e1600633 (doi:10.1126/sciadv.1600633) 27532052 10.1126/sciadv.1600633 PMC4980104

[39] Silcox MT, Dalmyn CK, Bloch JI 2009 Virtual endocast of *Ignacius graybullianus* (Paromomyidae, Primates) and brain evolution in early primates. Proc. Natl Acad. Sci. USA 106, 10 987–10 992. (doi:10.1073/pnas.0812140106) 19549862 10.1073/pnas.0812140106 PMC2708683

