# Supplementary material for: Oldest skeleton of a plesiadapiform provides additional evidence for an exclusively arboreal radiation of stem primates in the Palaeocene
Source: R Soc Open Sci. 2017 May 31;4(5):170329. doi: 10.1098/rsos.170329 (PMC5451839; doi:10.1098/rsos.170329)
Supplement: Electronic Supplementary Material: Oldest skeleton of a plesiadapiform provides evidence for an exclusively arboreal radiation of stem primates in the Paleocene [file rsos170329supp1.pdf]

Royal Society Open Science

Electronic Supplementary Material

Oldest skeleton of a plesiadapiform provides additional evidence for an exclusively arboreal radiation of stem primates in the Paleocene

Stephen G. B. Chester[a,b,c*], Thomas E. Williamson[d], Jonathan I. Bloch[e], Mary T. Silcox[f], Eric J. Sargis[g,h]

[a]Department of Anthropology and Archaeology, Brooklyn College, City University of New York, 2900 Bedford Avenue, Brooklyn, NY, 11210.
[b]Department of Anthropology, Graduate Center, City University of New York, 365 Fifth Avenue, New York, NY, 10016.
[c]New York Consortium in Evolutionary Primatology, New York, NY, 10024.
[d]New Mexico Museum of Natural History and Science, 1801 Mountain Road, NW, Albuquerque, NM 87104-1375
[e]Florida Museum of Natural History, University of Florida, 1659 Museum Road, Gainesville, FL, 32611-7800.
[f]Department of Anthropology, University of Toronto Scarborough, 1265 Military Trail, Scarborough, ON, M1C 1A4
[g]Department of Anthropology, Yale University, P. O. Box 208277, New Haven, CT, 06520
[h]Divisions of Vertebrate Paleontology and Vertebrate Zoology, Yale Peabody Museum of Natural History, New Haven, CT, 06520.

Correspondence to: Stephen G. B. Chester. Email: stephenchester@brooklyn.cuny.edu

This PDF File Includes:

Materials and Methods 2

- Institutional Abbreviations 2
- Phylogenetic Analysis 2

Geological setting and age of locality NMMNH L-6898 4

Electronic Supplementary Material References 6

Figures S1-S4 9

Tables S1-S2 15

Materials and Methods

Institutional Abbreviations

AMNH, American Museum of Natural History, New York, New York, U.S.A.; FMNH, Field Museum of Natural History, Chicago, Illinois, U.S.A.; MCZ, Museum of Comparative Zoology, Cambridge, Massachusetts, U.S.A.; NMMNH, New Mexico Museum of Natural History and Science, Albuquerque, New Mexico, U.S.A.; UCMP, University of California Museum of Paleontology, Berkeley, California, U.S.A.; UKMNH, University of Kansas Museum of Natural History, Lawrence, Kansas, U.S.A.; UM, University of Michigan Museum of Paleontology, Ann Arbor, Michigan, U.S.A.; UNSM, University of Nebraska State Museum, Lincoln, Nebraska, U.S.A.; USNM, United States National Museum of Natural History, Smithsonian Institution, Washington D.C., U.S.A.

Phylogenetic Analysis

The character matrix analyzed consists of 240 characters and 30 taxa (see Tables S1, S2) and was modified from that of [1], which was derived from previous analyses [2,3]; it was modified by adding the palaechthonids *Torrejonia wilsoni* and *Plesiolestes nacimienti*, and excluding apatemyids. The new partial skeleton of *T. wilsoni* (NMMNH P-54500) was scored into this matrix, and specimens of *Plesiolestes nacimienti* (UKMNH 9557, 9558, 9559; [4-6]) were scored into this matrix because this species is the only other palaechthonid known from more than dental fossils. Apatemyids introduced a considerable amount of missing data into the character matrix and acted as wildcard taxa. They were excluded from this dataset because they are not central to the focus of this study and their inclusion resulted in a less well-resolved hypothesis of relationships within Euarchontoglires. Additional specimens examined and

references used to score characters in the phylogenetic analysis can be found in appendix 5 of [2].

Maximum parsimony analysis was conducted using TNT (v1.5) software subsidized by the Willi Hennig Society [7], with all multistate characters treated as unordered. *Ukhaatherium* was designated as the outgroup. New Technology Search was implemented using 100 replications as the starting point for each hit, 200 parsimony ratchet iterations, 10 rounds of tree drifting, 10 rounds of tree fusing, and sectorial searching. The resulting most parsimonious tree (MPT) with a length of 997 steps was used as a starting tree in a Traditional Heuristic Search that was carried out using tree-bisection and reconnection (TBR). This resulted in a single most parsimonious tree with a consistency index (CI) of 0.318 and a retention index (RI) of 0.511 (Fig. S1). Absolute Bremer Support values were calculated using the suboptimal trees found in the search performed using the bremer.run script [8], which rigorously searches incrementally for suboptimal trees, with up to 10,000 trees held, increasing the score by 1 up to a maximum of 10 steps longer than the MPT (Fig. S1).

The same maximum parsimony analysis was conducted in TNT with a skeleton tree that forced Scandentia (*Ptilocercus* and *Tupaia*) outside a Primatomorpha grouping (*Cynocephalus* + Adapiforms + Omomyiforms):

```
(Ukhaatherium, (Ptilocercus_lowii, Tupaia_glis (Cynocephalus_volans,
Omomyidae, Adapidae)))
```

The two resulting MPTs with a length of 998 steps were used as starting trees in a Traditional Heuristic Search that was carried out using TBR. Two resulting MPTs were used to obtain a strict consensus (CI = 0.318, RI = 0.0510) (Fig. S2). Absolute Bremer Support values were

calculated using the suboptimal trees found in the search performed using the bremer.run script [8] (Fig. S2).

Geological setting and age of locality NMMNH L-6898

The partial skeleton of *Torrejonia wilsoni* (NMMNH P-54500) was found at locality NMMNH L-6898 in the Nacimiento Formation, San Juan Basin, New Mexico (Fig. S3). Locality NMMNH L-6898 is within the Torrejonian (To3) *Mixodectes pungens* zone of the West Flank of Torrejon Wash [9], which yields part of the type fauna for the Torrejonian NALMA [10,11]. This site is located within a normal polarity zone correlated with Chron C27n, which constrains the age of the site to about 62 Ma [12-14] (Fig. S4).

All of the fossil vertebrates at this locality were found in a small area of less than $1m^2$ of 2-3 cm thick massive muddy siltstone. Fossils were initially surface collected and then quarried from the deposit. All sediment from the quarry was processed using underwater screenwashing methods to recover the smallest bones. Some bones were articulated or associated in carbonate nodules and or encased in gypsiferous crusts. There appears to have been minimal transport before these bones were buried and this site might represent a small ephemeral pond. What is interpreted to be a partial skeleton of *Torrejonia wilsoni* was recovered mixed with partial skeletons of two other mammals [15], including a nearly complete skeleton of *Mixodectes pungens* (NMMNH P-54501) and a less complete skeleton of the eutherian mammal *Acmeodon secans* (Cimolestidae; NMMNH P-54499). Other associated fossil vertebrates include a partial skeleton of a neoavian land bird (Telluraves) [16,17], several undescribed partial skulls and postcrania of squamates, and numerous isolated mammal teeth [15].

Postcranial elements of *Torrejonia wilsoni* were readily distinguished from those of *Mixodectes pungens* by their smaller size. *Torrejonia wilsoni* is similar in size to *Acmeodon*

secans, but the morphology of many of the bones attributed to *T. wilsoni* closely resembles that of other dentally associated plesiadapiform skeletons. Also, although the skeleton of *Torrejonia* has a fully erupted adult dentition, many of the long bone epiphyses remain unfused, a pattern that has been previously documented for other plesiadapiforms (e.g. [18]). In contrast, epiphyseal fusion of postcranial elements attributed to *Acmeodon secans* indicates that this was a relatively more mature individual. For example, a right partial proximal femur attributed to *Acmeodon* has a fused greater trochanter, whereas the two associated proximal femora without proximal epiphyses were attributed to *Torrejonia* because they are very similar to those of other plesiadapiforms. Many additional elements such as fragmentary vertebrae, metapodials, and phalanges are more difficult to distinguish between these two mammals, so they are not considered further here. There are no duplicated tooth positions or skeletal elements for *T. wilsoni*, which suggests that only one individual of this species was present.

Supporting Information References

1. Bloch JI, Chester SGB, Silcox MT. 2016 Cranial morphology of Paleocene micromomyid plesiadapiform *Dryomomys szalayi* (Mammalia, Primates): implications for early primate evolution. *J. Hum. Evol.* **96**, 58-81. (doi:10.1016/j.jhevol.2016.04.001)
2. Silcox MT, Bloch JI, Boyer DM, Houde P. 2010 Cranial anatomy of Paleocene and Eocene *Labidolemur kayi* (Mammalia: Apatotheria), and the relationships of the Apatemyidae to other mammals. *Zool. J. Linn. Soc.* **160**, 773-825. (doi:10.1111/j.1096-3642.2009.00614.x)
3. Chester SGB, Bloch JI, Boyer DM, Clemens WA. 2015 Oldest known euarchontan postcrania and affinities of Paleocene *Purgatorius* to Primates. *Proc. Natl Acad. Sci. USA* **112**, 1487-1492. (doi:10.1073/pnas.1421707112)
4. Wilson RW, Szalay FS. 1972 New paromomyid primate from middle Paleocene beds, Kutz Canyon area, San Juan Basin. New Mexico. *Am. Mus. Novit.* **2499**, 1-18.
5. Kay RF, Cartmill M. 1977 Cranial morphology and adaptations of *Palaechthon nacimienti* and other Paromomyidae (Plesiadapoidea, ?Primates), with a description of a new genus and species. *J. Hum. Evol.* **6**, 19-53. (doi:10.1016/S0047-2484(77)80040-7)
6. Silcox MT, Williamson TE. 2012 New discoveries of early Paleocene (Torrejonian) primates from the Nacimiento Formation, San Juan Basin, New Mexico. *J. Hum. Evol.* **63**, 805-833. (doi:10.1016/j.jhevol.2012.09.002)
7. Goloboff PA, Catalano SA. 2016 TNT version 1.5, including a full implementation of phylogenetic morphometrics. *Cladistics* **32**, 221-238. (doi:10.1111/cla.12160)
8. Goloboff PA, Farris JS, Nixon KC. 2008 TNT, a free program for phylogenetic analysis. *Cladistics* **24**, 774-786. (doi:10.1111/j.1096-0031.2008.00217.x)

9. Williamson TE. 1996 The beginning of the age of mammals in the San Juan Basin, New Mexico; biostratigraphy and evolution of Paleocene mammals of the Nacimiento Formation. *New Mexico Mus. Nat. Hist. Sci. Bull.* **8**, 1-141.
10. Wood HE, Chaney RW, Clark JM, Colbert EH, Jepsen GL, Reeside Jr JR, Stock C. 1941 Nomenclature and correlation of the North American continental Tertiary. *Geol. Soc. Amer. Bull.* **52**, 1-48. (doi:10.1130/GSAB-52-1)
11. Lofgren DL, Lillegraven JA, Clemens WA, Gingerich PD, Williamson, TE. 2004 Paleocene biochronology: the Puercan through Clarkforkian land mammal ages. *Late Cretaceous and Cenozoic mammals of North America* (ed MO Woodburne), pp. 43-105. New York: Columbia University Press.
12. Kuiper KF, Deino A, Hilgen FJ, Krijgsman W, Renne PR, Wijbrans JR. 2008 Synchronizing rock clocks of Earth history. *Science* **320**, 500-504. (doi:10.1126/science.1154339)
13. Luterbacher HP, et al. 2004 The Paleogene Period. *A Geologic Time Scale 2004* (eds FM Gradstein, JG Ogg, AG Smith), pp. 384-408. Cambridge: Cambridge University Press.
14. Ogg JG. 2012 Geomagnetic polarity time scale. *The Geologic Time Scale 2012* (eds FM Gradstein, JG Ogg, MD Schmitz, G Ogg), pp. 85-113. Oxford: Elsevier.
15. Williamson TE. 2007 Exceptional preservation of mammals, a bird, and lizards from the lower Paleocene of New Mexico. *J. Vert. Paleontol.* **27**, 166A.
16. Stidham TA, Williamson TE. 2007 A unique derived possibly zygodactyl bird skeleton from the Paleocene (Torrejonian) of New Mexico. *J. Vert. Paleontol.* **27**,152A.

17. Ksepka DT, Stidham TA, Williamson TE. 2015 A new species of early Paleocene landbird and the post-Cretaceous diversification of birds in North America. *J. Vert. Paleontol. Program and Abstracts*, 159.
18. Chester SGB, Bloch JI. 2013 Systematics of Paleogene Micromomyidae (Euarchonta, Primates) from North America. *J. Hum. Evol.* **65**, 109-142. (doi:10.1016/j.jhevol.2013.04.006)
19. Taylor LH, Butler RF. 1980 Magnetic-polarity stratigraphy of Torrejonian sediments, Nacimiento Formation, San Juan Basin, New Mexico. *Am. J. Sci.* **280**, 97-115. (doi:10.2475/ajs.280.2.97)
20. MacPhee RDE, Novacek MJ, Storch G. 1988 Basicranial morphology of early Tertiary erinaceomorphs and the origin of Primates. *Am. Mus. Novit.* **2921**, 1-42.
21. Wible JR. 2008 On the cranial osteology of the Hispaniolan solenodon, *Solenodon paradoxus* Brandt, 1833 (Mammalia, Lipotyphla, Solenodontidae). *Ann. Carnegie Mus.* **77**, 321-402. (doi:10.2992/0097-4463-77.3.321)
22. Novacek MJ. 1986 The skull of leptictid insectivorans and the higher-level classification of eutherian mammals. *Bull. Am. Mus. Nat. Hist.* **183**, 1-112.
23. McDowell SB Jr. 1958 The Greater Antillean insectivores. *Bull. Am. Mus. Nat. Hist.* **115**, 117-214.
24. Silcox MT. 2001 A phylogenetic analysis of Plesiadapiformes and their relationship to Euprimates and other archontans. Ph.D. Dissertation, Johns Hopkins University School of Medicine.

Figure S1. Hypothesis of evolutionary relationships of *Torrejonia wilsoni* and other eutherian mammals.

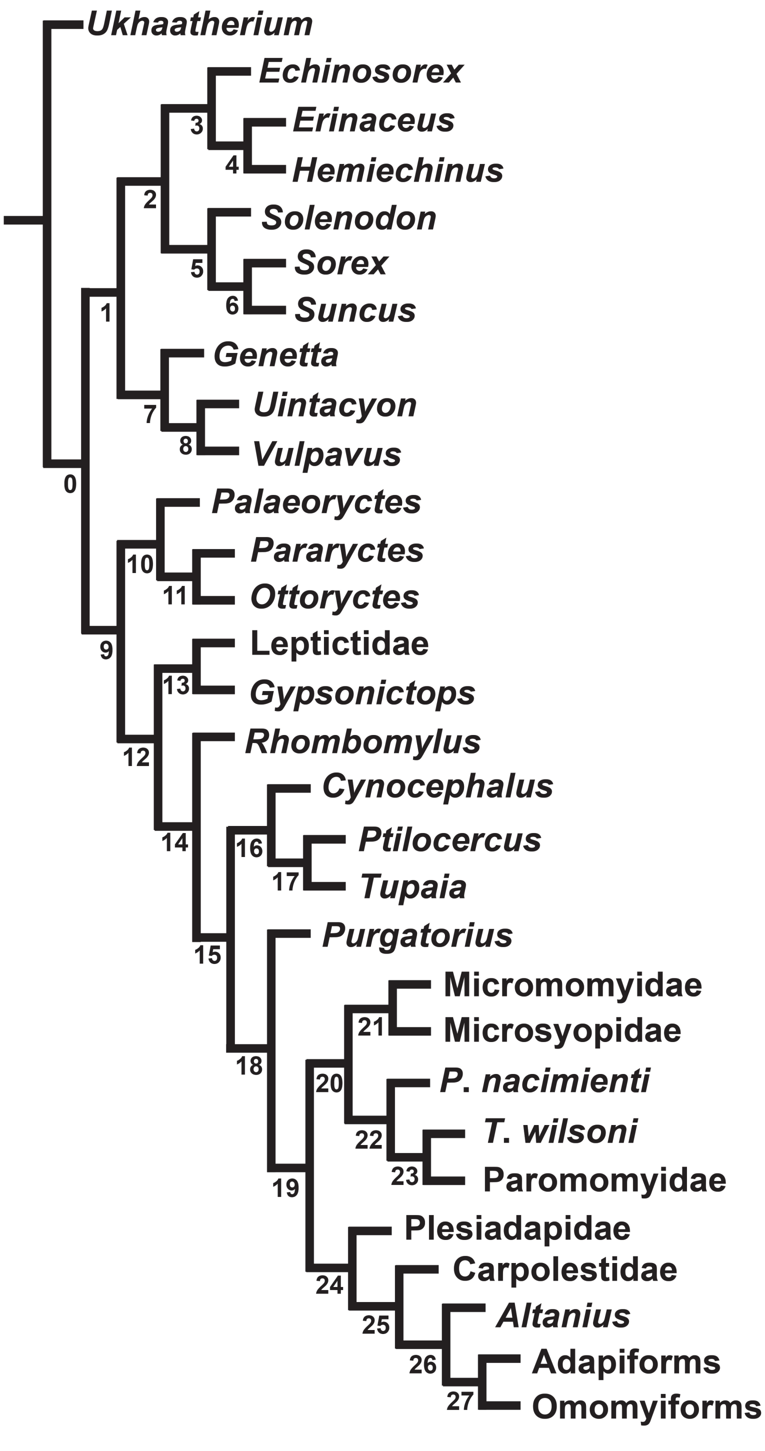

Figure S1. Hypothesis of evolutionary relationships of *Torrejonia wilsoni* and other eutherian mammals. Results of cladistic analysis based on 30 taxa and 240 morphological characters (68 postcranial, 45 cranial, 127 dental; Table S1). Resulting single most parsimonious cladogram with Bremer support indices calculated in TNT for listed nodes are as follows: **0(ingroup)**; **1(Laurasiatheria)** = 4; **2(Eulipotyphla)** = 6; **3(Erinaceomorpha)** = 12; **4** = 4; **5(Soricomorpha *sensu lato*)** = 4; **6(Soricidae)** = 17; **7(Carnivora)** = 3; **8** = 1; **9** = 1; **10(Palaeoryctidae)** = 5; **11** = 2; **12** = 1; **13(Leptictida)** = 2; **14(Euarchontoglires)** = 2; **15(Euarchonta)** = 3; **16(Sundatheria)** = 1; **17(Scandentia)** = 3; **18(Primates)** = 2; **19** = 1; **20** = 1; **21** = 4; **22(Paromomyoidea)** = 1; **23** = 1; **24(Euprimateformes)** = 1; **25** = 1; **26(Euprimates)** = 6; **27** = 1. Unambiguous synapomorphies calculated in TNT for listed nodes are as follows: **14(Euarchontoglires)** = 105(2), 135(1), 151(1), 158(1), 167(0), 172(1), 229(1); **15(Euarchonta)** = 11(1), 29(0), 33(0), 37(0), 40(0), 45(2), 130(0), 145(0), 207(1), 217(1); **16(Sundatheria)** = 19(1), 58(1), 142(1), 160(2), 189(0), 212(0), 221(2), 229(0), 235(3); **17(Scandentia)** = 39(1), 42(0), 52(1), 66(2), 72(2), 105(1), 129(0), 144(1), 149(1), 150(1), 153(1), 165(2), 168(0), 170(1), 175(1), 194(1), 204(1), 205(3), 218(1); **18(Primates)** = 115(1), 185(0), 201(0), 205(1); **19** = 166(1), 179(1), 190(1), 194(1); **20** = 21(1), 71(0), 88(1), 91(1), 92(1), 128(1), 132(0), 168(0), 200(1); **21** = 103(0), 121(1), 135(0), 172(0), 189(2), 192(1), 210(1), 221(1), 228(3); **22(Paromomyoidea)** = 213(1), 224 (1), 234(1); **23** = 235(2), 237(1); **24(Euprimateformes)** = 56(1), 75(0), 101(1), 143(1), 159(1), 209(1), 212(0), 214(1), 215(1), 218(1); **25** = 9(1), 12(1), 21(2), 27(1), 55(1), 57(1), 122(0), 170(1), 201(1); **26(Euprimates)** = 129(0), 144(1), 147(0), 149(1), 166(0), 171(1), 173(2), 175(1), 185(1), 187(0), 193(0), 232(0) 235(2); **27** = 218(0), 227(0).

Figure S2. Hypothesis of evolutionary relationships of *Torrejonia wilsoni* and other eutherian mammals with Primatomorpha enforced.

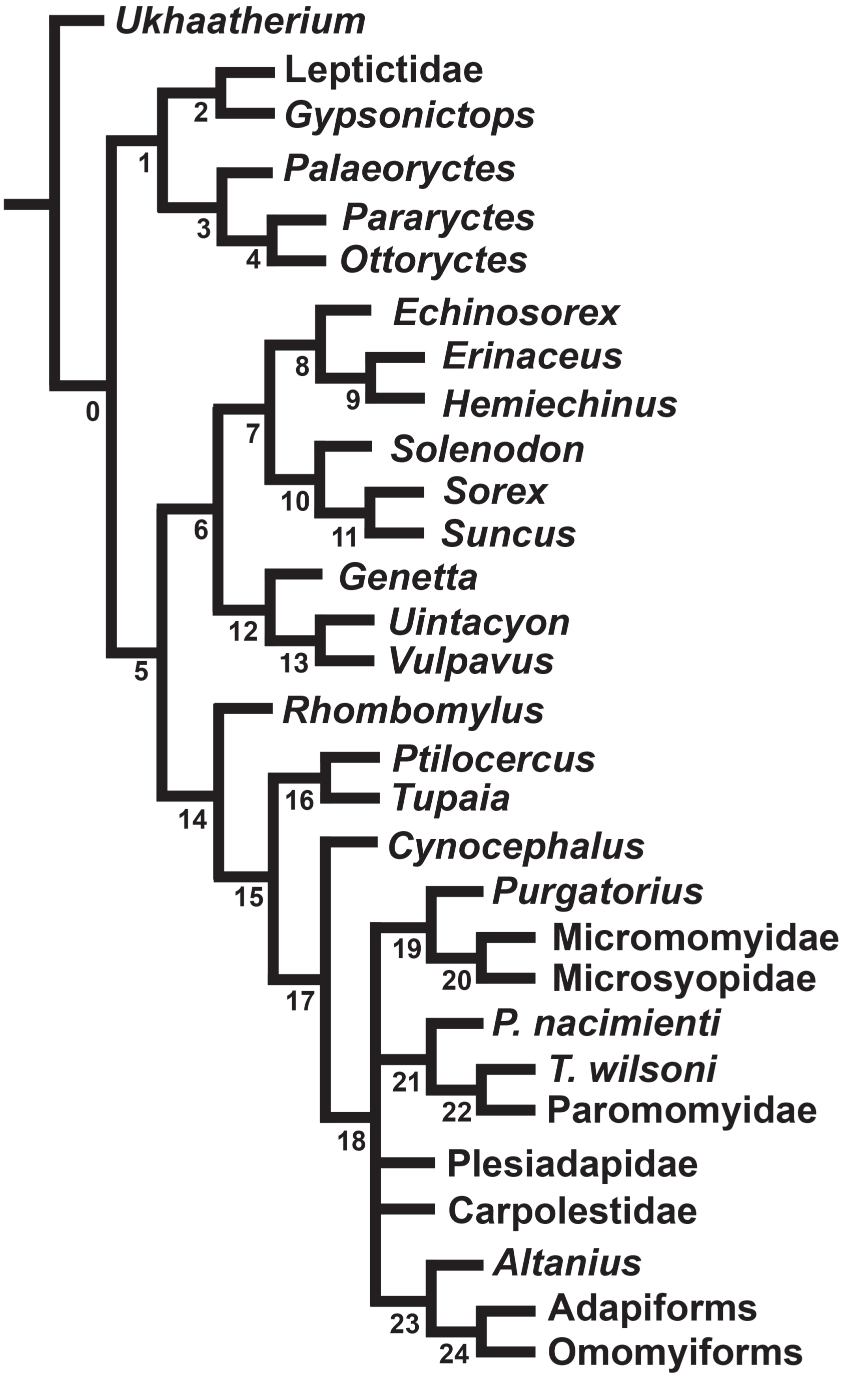

Figure S2. Hypothesis of evolutionary relationships of *Torrejonia wilsoni* and other eutherian mammals with Primatomorpha enforced. Results of cladistic analysis based on 30 taxa and 240 morphological characters (68 postcranial, 45 cranial, 127 dental; Table S1) with a skeleton tree that forced Scandentia (*Ptilocercus* and *Tupaia*) outside a Primatomorpha grouping (*Cynocephalus* + Adapiforms + Omomyiforms). Resulting strict consensus of two most parsimonious trees with Bremer support indices calculated in TNT for listed nodes are as follows: **0(ingroup)**; **1** = 1; **2(Leptictida)** = 2; **3(Palaeoryctidae)** = 5; **4** = 2; **5** = 4; **6(Laurasiatheria)** = 6; **7(Eulipotyphla)** = 15; **8(Erinaceomorpha)** = 4; **9** = 1; **10(Soricomorpha sensu lato)** = 4; **11(Soricidae)** = 19; **12(Carnivora)** = 3; **13** = 1; **14(Euarchontoglires)** = 2; **15(Euarchonta)** = 1; **16(Scandentia)** = 3; **17(Primatomorpha)** = 3; **18(Primates)** = 1; **19** = 2; **20** = 1; **21(Paromomyoidea)** = 1; **22** = 1; **23(Euprimates)** = 1; **24** = 4. Unambiguous synapomorphies calculated in TNT for listed nodes are as follows: **14(Euarchontoglires)** = 42(1), 69(1), 120(1), 122(1), 135(1), 151(1), 158(1), 193(1), 208(0), 233(1); **15(Euarchonta)** = 29(0), 33(0), 40(0), 45(2), 76(1), 130(0), 145(0), 197(1); **16(Scandentia)** = 39(1), 42(0), 52(1), 66(2), 72(2), 105(1), 149(1), 153(1), 168(0), 175(1), 199(3); **17(Primatomorpha)** = 51(1), 81(1), 163(0), 165(0), 227(1), 231(2); **18(Primates)** = 73(0), 78(1), 107(1), 156(0), 179(1), 219(0), 226(1), 228(2), 229(1); **19** = 116(2), 135(0), 172(0), 176(1), 227(0), 228(1), 231(01); **20** = 189(2), 192(1), 210(1), 220(1), 221(1), 229(3); **21(Paromomyoidea)** = 213(1), 224(1), 234(1); **22** = 206(1), 235(2), 237(1); **23(Euprimates)** = 147(0), 149(1), 175(1), 187(0), 193(0); **24** = 206(1), 227(0).

Figure S3. Map showing the location of NMMNH fossil locality L-6898 in relation to the outcrop belt of the Nacimiento Formation, San Juan Basin, New Mexico.

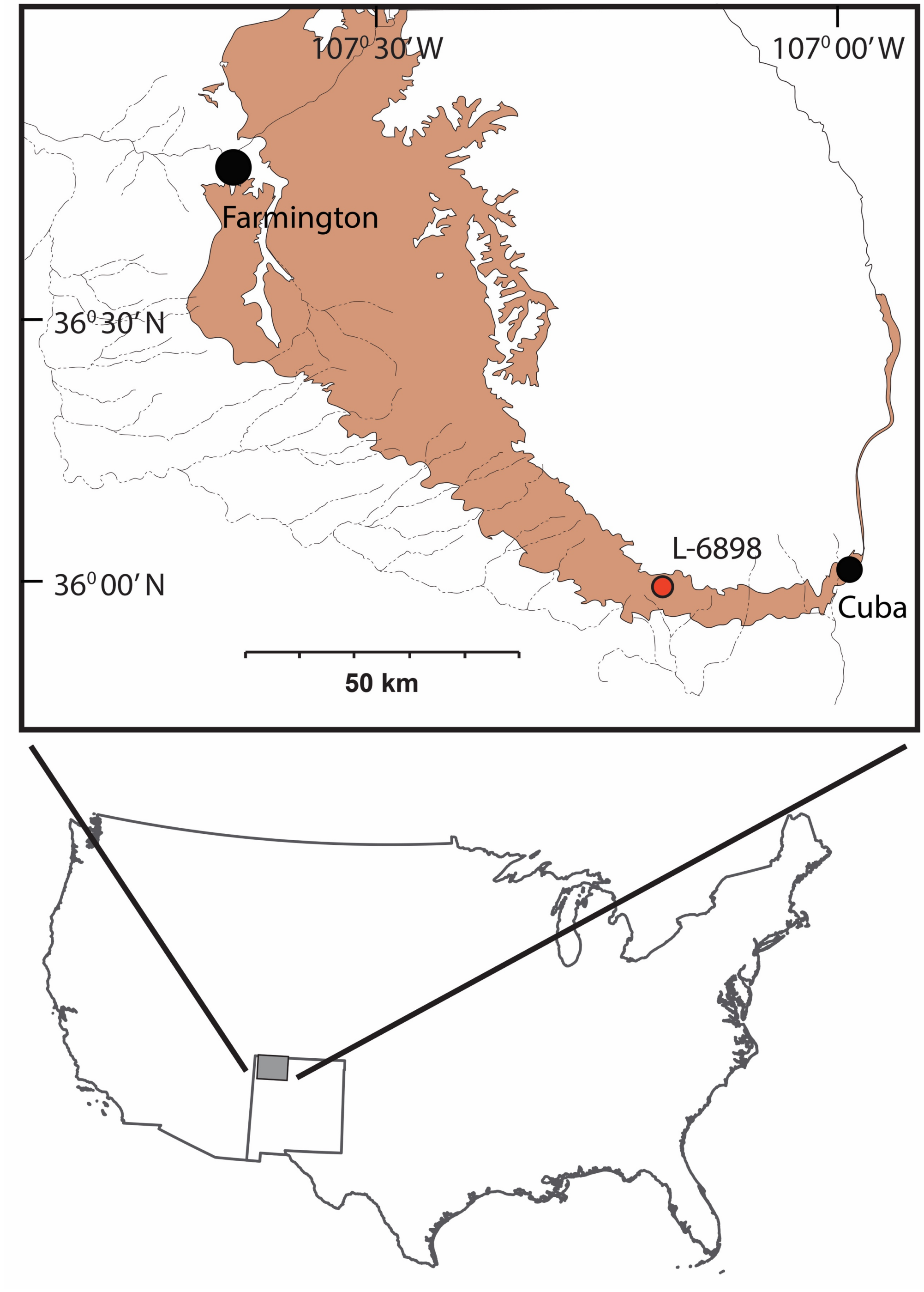

Figure S4. Stratigraphic section of a part of the Nacimiento Formation as measured at the West Flank of Torreon Wash showing the position of NMMNH locality L-6898 relative to marker beds and magnetic polarity zonations [9, 19]. The stratigraphic section is correlated to Nacimiento Formation biostratigraphic zonation [9], mammal biochronology (Paleocene North American Land Mammal Ages [NALMA] and interval zones; ref. 11), and the geomagnetic polarity time scale (GPTS; ref. 14). The precise ages of the polarity reversals at the bottom and top of chron C27n are from [14].

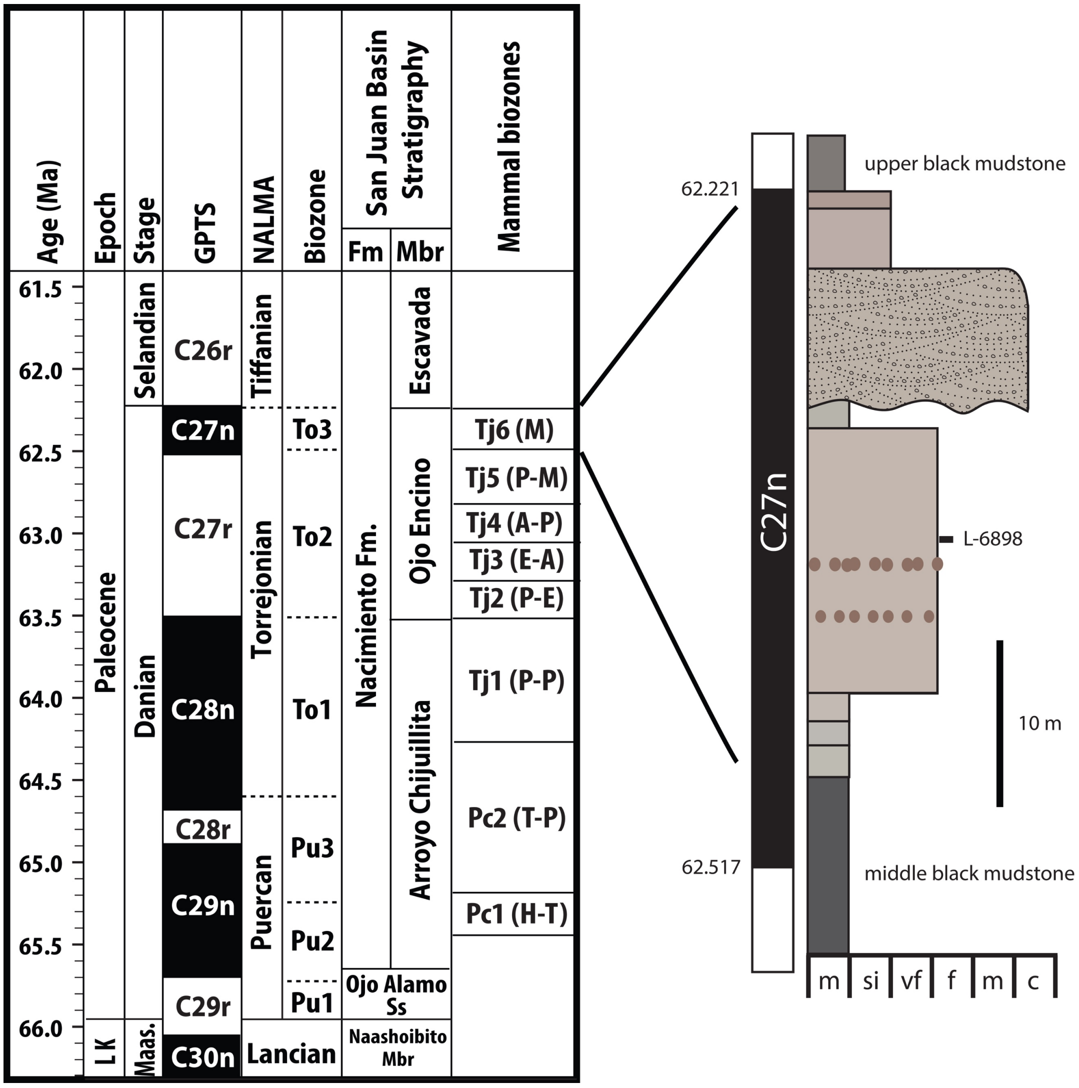

Supplementary Tables

Table S1. Descriptions of morphological characters used in the cladistic analysis modified from that of [1], which was derived from previous analyses [2, 3]. Each character is listed with one or two letters that refer to the larger partition to which they belong (i.e., PC = postcranial; Cr = cranial; D = dental).

1. PC, Scapula, Metacromion form: (0) absent; (1) small but present; (2) very large - larger than acromion.

2. PC, Humerus, Projection of greater tuberosity: (0) small tuberosity that does not extend superior to head; (1) even with or slightly above head; (2) extends far superior to head.

3. PC, Humerus, Projection of lesser tuberosity: (0) not projecting, small; (1) large and medially projecting.

4. PC, Humerus, Deltopectoral crest form: (0) sharp and elevated; (1) broad and elevated.

5. PC, Humerus, Deltopectoral crest tip form: (0) crest tapers to a point; (1) crest has broad, rounded, shelf-like distal end.

6. PC, Humerus, Deltopectoral crest proportional length: (0) less than 50% humerus length; (1) between 50% and 67% humerus length: (2) greater than 67% humerus length.

7. PC, Humerus, Olecranon fossa depth: (0) shallow, or slit-like; (1) deep and pit-like; (2) deep, pit-like, and perforated.

8. PC, Humerus, Medial epicondyle projection: (0) makes up less than 25% of entire distal end width; (1) makes up 25% or more of entire distal end width.

9. PC, Humerus, Supinator crest development: (0) present as a distinct ridge; (1) projects prominently posterolaterally; (2) absent - rounded lateral surface of distal humeral shaft.

10. PC, Humerus, Prominence of teres tubercle on medial side: (0) small or indistinct; (1) prominent and crest-like.

11. PC, Humerus, Capitulum shape: (0) spindle-shaped; (1) ovoid or spherical.

12. PC, Humerus, Humeral trochlear morphology: (0) medial keel only; (1) medial and lateral keels, trochlea and capitulum well-separated.

13. PC, Radius, Radial head shape: (0) minimum diameter greater than 70% maximum diameter; (1) minimum diameter between 70% and 60% maximum diameter; (2) minimum diameter less than or equal to 60% maximum diameter.

14. PC, Radius, Bicipital tuberosity presence: (0) present; (1) absent.

15. PC, Radius, Distal radius-ulna contact: (0) ligamentous or synovial; (1) synostosis.

16. PC, Radius, Ridge on dorsal surface of distal end presence: (0) absent; (1) present.

17. PC, Ulna, Olecranon relative length: (0) less than 20% total ulna length; (1) between 20% and 25% of total ulna length; (2) greater than 25% total ulna length.

18. PC, Ulna, Olecranon tip form: (0) straight, with no flare beyond more proximal part of olecranon; (1) tip flares somewhat medially; (2) tip flares prominently medially.

19. PC, Carpals, Scaphoid-lunate fusion: (0) unfused; (1) fused.

20. PC, Metapodial, MCIII dorsal surface form: (0) smooth; (1) with distinct extensor tubercle.

21. PC, Phalanges, Digit elongation index ([Intermediate phalanx length + proximal phalanx length]/ humerus length) of digit III or IV: (0) less than 35%; (1) 35%-50%; (2) greater than 50%.

22. PC, Phalanges, Prehensility index of digit III of the manus: (0) intermediate phalanx less than 80% of metacarpal length; (1) greater than or equal to 80%.

23. PC, Innominate, Anterior inferior iliac spine development: (0) absent; (1) distinct but small; (2) pronounced and laterally projecting.

24. PC, Innominate, Ilium shape: (0) rod-like; (1) blade-like.

25. PC, Innominate, Buttressing of acetabulum: (0) no buttressing; (1) cranial buttressing; (2) caudal buttressing.

26. PC, Innominate, Ischiopubic symphysis presence and form: (0) absent; (1) present but narrow craniocaudally (2) robust - long craniocaudally.

27. PC, Femur, Greater trochanter projection: (0) below femoral head (ratio of femoral length including greater trochanter to that length not including trochanter, but measured to the superior surface of the head, is less than 1); (1) even with femoral head (ratio is between 1 and 1.05); (2) prominent, extending above femoral head (ratio is greater than 1.05).

28. PC, Femur, Greater trochanter relative anteroposterior (AP) expansion: (0) trochanter AP dimension less than 120% midshaft AP dimension; (1) trochanter AP dimension 120% or greater midshaft AP dimension.

29. PC, Femur, Lesser trochanter orientation: (0) medially projecting; (1) posteromedially or posteriorly projecting.

30. PC, Femur, Third trochanter position: (0) far distal to lesser trochanter; (1) slightly distal to lesser trochanter; (2) proximal to lesser trochanter.

31. PC, Femur, Third trochanter lateral projection: (0) small, not projecting; (1) prominently projecting.

32. PC, Femur, Patellar groove form: (0) proximodistal length less than 150% of mediolateral dimension; (1) proximodistal length greater than or equal to 150% of mediolateral dimension.

33. PC, Tibia-Fibula, Relative shaft length: (0) tibia no longer than femur; (1) tibia longer than femur.

34. PC, Tibia-Fibula, Tibia shaft shape: (0) straight; (1) bowed and thus laterally concave.

35. PC, Tibia-Fibula, Popliteal tuberosity (process on the anteromedial surface of the proximal tibia) presence: (0) present; (1) absent.

36. PC, Tibia-Fibula, Distal contact form: (0) ligamentous or synovial; (1) synostosis - fused.

37. PC, Tibia-Fibula, Tibial posterior process development: (0) small or absent; (1) prominently distally projecting.

38. PC, Tibia-Fibula, Medial malleolus form: (0) well-developed; (1) small; (2) absent.

39. PC, Tibia-fibula, Medial malleolus relation to sustentaculum tali (scored as inapplicable if medial malleolus was scored as "absent" in the preceding character): (0) no posterior contact; (1) posterior contact.

40. PC, Astragalus, Form of trochlea of body: (0) shallowly grooved; (1) deeply grooved.

41. PC, Astragalus, Regions of trochlea of body: (0) not clearly separated into regions or regions equal in mediolateral width; (1) lateral region wider than medial region; (2) medial region wider than lateral region.

42. PC, Astragalus, Relative height of trochlear borders: (0) medial border less than 90% height of lateral border; (1) medial border 90% to 110% height of lateral border; (2) medial border greater than 110% height of lateral border.

43. PC, Astragalus, Astragalar body medial aspect: (0) flat; (1) deeply concave, cotylar fossa.

44. PC, Astragalus, Astragalar medial border of body crimped (medial margin is relatively deeper dorsoventrally than long proximodistally): (0) absent; (1) present.

45. PC, Astragalus, Sustentacular and navicular facet contact: (0) no contact; (1) contact on lateral side; (2) contact on medial side; (3) contact on ventral side.

46. PC, Astragalus, Fibular facet form and orientation: (0) flat and faces laterodorsally; (1) faces laterally but has laterally-flaring, dorsally-facing shelf; (2) flat and faces laterally with no shelf.

47. PC, Astragalus, Ectal facet form (0) evenly concave, (1) unevenly concave or "peaked."

48. PC, Astragalus, Head shape: (0) maximum diameter less than 140% of minimum diameter; (1) greater than or equal to 140%.

49. PC, Astragalus, Flexor fibularis groove presence: (0) present, separate from trochlea; (1) absent.

50. PC, Calcaneum, Fibular facet orientation: (0) large and lateral; (1) large and distal; (2) small or absent.

51. PC, Calcaneum, Plantar pit on cuboid facet presence: (0) absent; (1) present.

52. PC, Calcaneum, Peroneal tubercle position: (0) distal; (1) proximal.

53. PC, Calcaneum, Ectal facet proximal margin shape: (0) convex; (1) concavoconvex.

54. PC, Calcaneum, Shaft (body and tuber) shape: (0) straight or laterally bowed; (1) medially bowed (= laterally convex).

55. PC, Entocuneiform, Plantodistal process presence: (0) present; (1) absent.

56. PC, Entocuneiform, Proximal extension of medial metatarsal 1 facet presence: (0) absent; (1) present.

57. PC, Metapodial, Metatarsal I torsion: (0) absent; (1) present.

58. PC, Metapodial, Bifurcate keel on metatarsal I presence: (0) absent; (1) present.

59. PC, Metapodial, Cylindrical (instead of spherical) metapodial heads presence (score from the central metapodials of either the manus or pes): (0) absent; (1) present.

60. PC, Phalanges, Distal phalanx of pedal digit I shape: (0) claw shaped; (1) flattened as a nail.

61. PC, Phalanges, Ungual pedal phalanx of digit III-IV relative length: (0) greater than 110% length of respective intermediate phalanges; (1) less than or equal to 110%.

62. PC, Phalanges, Asymmetrical manual or pedal intermediate phalanx distal ends presence: (0) absent; (1) present.

63. PC, Phalanges, Flexor sheath attachments on proximal phalanges of the manus or pes: (0) reduced or present as bony processes; (1) present as long ridges but not substantially ventrally projecting; (2) substantially ventrally projecting.

64. PC, Axial, Axis spinous process orientation: (0) caudal; (1) cranial.

65. PC, Axial, Anapophysis number: (0) present on all or all but ultimate lumbar vertebrae; (1) lacking on all or all but first lumbar vertebrae.

66. PC, Axial, Sacral vertebra spinous process: (0) all equal; (1) first reduced or absent; (2) first two reduced or absent.

67. PC, Axial, Manubrium sterni form: (0) not enlarged; (1) enlarged with ventral keel that extends to anterior margin; (2) dorsoventrally thickened with ventral keel poorly developed; (3) with prominent anterior process and short posterior process.

68. PC, Axial, Rib morphology: (0) narrow; (1) broad.

69. Cr, Snout, Length: (0) long; (1) moderate; (2) very short.

70. Cr, Nasal, Posterior extension: (0) extends to M^3; (1) extends to M^1; (2) extends to P^4.

71. Cr, Nasal, Frontonasal contact relative size: (0) broad, nasals reach lacrimal and maxilla is separated from frontal; (1) semi expanded - nasals flare posteriad but do not touch lacrimal; (2) restricted - nasals narrow posteriad.

72. Cr, Jugal, Zygomatic arch form: (0) incomplete - jugal absent; (1) complete without postorbital bar; (2) complete with postorbital bar.

73. Cr, Lacrimal, Tubercle development: (0) distinctly present; (1) poorly defined or absent.

74. Cr, Premaxilla, Frontal contact: (0) absent; (1) present.

75. Cr, Maxilla, Orbital mosaic maxillary contacts: (0) frontal; (1) palatine excludes from frontal contact; (2) non-palatine bone prevents maxillary-frontal contact.

76. Cr, Maxilla, Infraorbital foramen size: (0) larger than 15% maximum breadth between cheek tooth arcades; (1) 15% or less.

77. Cr, Maxilla, Infraorbital foramen position: (0) above M^1; (1) above P^4; (2) above P^3.

78. Cr, Palatine, Postpalatine spine presence: (0) large; (1) small or absent.

79. Cr, Alisphenoid, Tympanic process development: (0) small or absent; (1) substantial - may form anterior margin of bulla.

80. Cr, Alisphenoid, Ectopterygoid crest development: (0) no alisphenoid-tipped ectopterygoid crest; (1) alisphenoid-tipped crest equal or smaller than entopterygoid crest; (2) alisphenoid-tipped crest much larger than entopterygoid crest.

81. Cr, Alisphenoid, Canal for ramus infraorbitalis presence: (0) present; (1) absent.

82. Cr, Alisphenoid, Foramen rotundum presence: (0) present; (1) absent.

83. Cr, Alisphenoid, Borders of foramen ovale: (0) foramen ovale contained by alisphenoid; (1) between alisphenoid and squamosal and/or petrosal.

84. Cr, Alisphenoid, Transverse canal (foramen subovale) presence: (0) absent; (1) present.

85. Cr, Basisphenoid, Tympanic process development: (0) small to absent; (1) substantial - may form much of anteromedial wall and floor of bulla.

86. Cr, Basisphenoid, Morphology relating to vidian nerve: (0) foramen for vidian nerve in basisphenoid in tympanic cavity; (1) groove leads to foramen outside of tympanic cavity; (2) no morphological evidence of vidian nerve.

87. Cr, Basisphenoid, Anterior carotid foramen composition: (0) piriform fenestra; (1) basisphenoid.

88. Cr, Basioccipital, Tympanic process development: (0) absent; (1) distinctly present.

89. Cr, Basioccipital, Central stem breadth: (0) mediolaterally broad central stem, tympanic cavities well separated; (1) mediolaterally narrow central stem, tympanic cavities nearly in contact.

90. Cr, Basioccipital, Dorsum sellae presence: (0) absent; (1) present, with prominent posterior clinoid processes.

91. Cr, Occipital, Tentorium cerebelli condition: (0) unossified; (1) ossified.

92. Cr, Occipital, Nuchal crest development: (0) poorly developed or absent; (1) distinct and large.

93. Cr, Squamosal, Postglenoid process form: (0) absent; (1) present rostral to postglenoid foramen; (2) present lateral or caudal to postglenoid foramen.

94. Cr, Squamosal, Entoglenoid process form: (0) absent; (1) present but small (smaller than postglenoid process - if present); (2) present and large.

95. Cr, Squamosal, Pathway for the ramus inferior of the stapedial artery location: (0) separate from chorda tympani nerve and Glaserian fissure; (1) within Glaserian fissure with chorda tympani nerve.

96. Cr, Squamosal/Petrosal, Epitympanic recess size: (0) small (less than half the width of the promotorium); (1) large (more than half the width of the promontorium).

97. Cr, Ectotympanic, Degree to which it is covered by other bones: (0) phaneric; (1) completely covered by bony bulla.

98. Cr, Ectotympanic, Shape: (0) very narrow ring; (1) moderately expanded ring; (2) vastly expanded - may form much or all of ossified bulla.

99. Cr, Petrosal, Caudal tympanic process development: (0) absent or very small; (1) present and not very small (small to extensive *sensu* ref. 20).

100. Cr, Petrosal, Rostral tympanic process presence: (0) small or absent; (1) present.

101. Cr, Petrosal, Bulla presence (score as inapplicable if rostral tympanic process is absent): 0 absent; (1) present.

102. Cr, Petrosal, Piriform fenestra development: (0) expansive medially, laterally, and/or caudally; (1) reduced or absent

103. Cr, Petrosal, Facial nerve pathway: (0) open sulcus; (1) fully closed canal.

104. Cr, Petrosal, Expression of promontory branch of ICA on promontorium: (0) groove; (1) tube; (2) no expression.

105. Cr, Petrosal, Stapedial branch of internal carotid artery (ICA) on promontorium expression: (0) distinct groove; (1) tube; (2) little or no expression.

106. Cr, Petrosal Bony tube for stapedial artery condition (1.0) (score as inapplicable if bony tube is absent): (0) stops at fenestra vestibule; (1) continues through fenestra vestibuli

107. Cr, Petrosal, Fenestra cochleae visibility: (0) visible (when bulla, if present, is removed); 1 shielded by petrosal.

108. Cr, Entotympanic, Presence and degree of development: (0) absent; (1) present, small; (2) present and contributes to much of an ossified bulla.

109. Cr, Parietal/Occipital, Foramina in the lateral braincase (likely for the rami temporales; ref. 21; =sinus canal; ref. 22) presence: (0) absent; (1) one or two present; (2) many present, proliferated.

110. Cr, Parietal, Temporal lines or crest form: (0) single sagittal crest; (1) parallel parasagittal crests or temporal lines.

111. Cr, Parietal, Orbitotemporal canal (*sensu* ref. 21; =sinus canal, ref. 23; =ophthalmic sulcus, ref. 22) for ramus supraorbitalis of ramus superior presence: (0) present - groove on internal aspect of braincase; (1) absent.

112. Cr, Dentary, Internal ridge caudal to toothrow development: (0) incomplete, reduced or absent ridge between M_3 and condyle; (1) prominent ridge between M_3 and condyle.

113. Cr, Dentary, Posteriormost mental foramen position: (0) beneath m1 or farther distal; (1) beneath P_4; (2) beneath P_3 or farther mesial.

114. D, I^1, Size: (0) similar in size to other incisors or premolars (if I^{2-3} are missing); (1) much larger than other incisors or premolars (if I^{2-3} are missing); (2) tooth absent.

115. D, I^1, Tip strongly recurved presence: (0) absent; (1) present.

116. D, I^1, Accessory cuspules presence: (0) no accessory cuspules; (1) posterocone present but no apical cuspules; (2) posterocone and small cuspules developed around the tip; no strong apical division; (3) strong apical division into an anterocone and laterocone in addition to the presence of a protocone.

117. D, I^1, Restricted enamel presence and distribution: (0) absent (enamel surrounds the entire tooth); (1) restricted to an anterior band; (2) bands of enamel present on both anterior and posterior surfaces.

118. D, I^2, Presence: (0) present; (1) absent.

119. D, I^2, Size: (0) tooth absent; (1) present but small (2) large.

120. D, I^3, Presence: (0) present; (1) absent.

121. D, C^1, Upper canine root number: (0) single rooted; (1) double rooted; (2) tooth absent.

122. D, P^1, Presence: (0) present; (1) absent.

123. D, P^2, Root number: (0) tooth absent; (1) double rooted; (2) single rooted, (3) triple rooted.

124. D, P^2, Parastyle (anterior basal cusp) presence: (0) poorly developed or absent; (1) distinct.

125. D, P^3, Root number: (0) triple rooted; (1) double rooted; (2) single rooted; (3) tooth absent.

126. D, P^3, Shape (buccal length/lingual length): (0) less than 1.8; (1) 1.8-2.0; (2) more than 2.0.

127. D, P^3, Size relative to P^4 based on (ln(buccal length x width)P^3)/(ln(buccal length x width)P^4): (0) less than 0.6; (1) 0.6-1.2; (2) more than 1.2.

128. D, P^3, Parastyle (anterior basal cusp) presence: (0) distinct; (1) absent.

129. D, P^3, Metacone presence: (0) absent; (1) present.

130. D, P^3, Metastyle presence: (0) absent; (1) present.

131. D, P^3, Conules presence and number: (0) absent; (1) one present; (2) two present.

132. D, P^3, Protocone presence: (0) absent; (1) present.

133. D, P^3, Hypocone presence: (0) absent; (1) present.

134. D, P^4, Number of roots: (0) one; (1) two; (2) three.

135. D, P^4, Shape (buccal length/lingual length): (0) more than 1.8; (1) less than or equal to 1.8.

136. D, P^4, Cusp acuteness: (0) acute; (1) bulbous.

137. D, P^4, Carnassial shear with m1 presence: (0) absent; (1) present.

138. D, P^4, Size relative to M^1 based on (ln(buccal length x width)P^4)/(ln(buccal length x width)M^1): (0) less than 0.9; (1) 0.90-0.98; (2) more than 0.98.

139. D, P^4, Width relative to M^1: (0) P^4 not as wide transversely as M^1; (1) P^4 as wide as or wider transversely as M^1.

140. D, P^4, Cusp height in lateral view relative to M^1: (0) P^4 lower than M^1; (1) P^4 equal to or greater in height than M^1.

141. D, P^4, Stylar shelf development: (0) wide laterally and very narrow in the middle because of a strong ectoflexus; (1) ectoflexus weak with little or no stylar shelf.

142. D, P^4, Parastyle presence: (0) present; (1) absent.

143. D, P^4, Parastylar lobe morphology: (0) large, projecting; (1) small, not projecting.

144. D, P^4, Metacone presence: (0) present; (1) absent.

145. D, P^4, Metastyle presence: (0) absent; (1) present.

146. D, P^4, Conules presence and size: (0) absent; (1) one large conule present located near the midline of the tooth mesiodistally; (2) small paraconule present; (3) both conules present, strong; (4) metaconule present, no paraconule.

147. D, P^4, Protocone lobe shape: (0) shorter mesiodistally than wide; (1) equally long and wide.

148. D, P^4, Preprotocrista presence: (0) present; (1) absent.

149. D, P^4, Protocone position: (0) not mesial to paracone; (1) mesial to paracone.

150. D, P^4, Postprotocrista presence: (0) present; (1) absent.

151. D, P^4, Postprotocingulum presence: (0) absent; (1) present.

152. D, P^4, Hypocone presence: (0) totally absent; (1) present, at least incipiently.

153. D, M^1, Length relative to transverse width compared to M^2 or M^3: (0) M^1 similarly elongate relative to transverse width than M^2 or M^3; (1) M^1 more elongate relative to transverse width than M^2 or M^3.

154. D, M^1, Ectoflexus depth: (0) deep with the stylar shelf wide at the corners and almost disappearing in the middle; (1) shallow.

155. D, M^1, W-shaped ectoloph presence: (0) absent; (1) present.

156. D, M^1, Precingulum presence: (0) present, doesn't connect to postcingulum; (1) present, connects to postcingulum in at least some specimens; (2) precingulum absent.

157. D, M^1, Pre- and paracingula continuity: (0) not continuous; (1) continuous; (2) no paracingulum.

158. D, M^1, Parastylar lobe morphology: (0) projecting beyond the plane of the mesiolingual corner of the tooth; (1) not projecting.

159. D, M^1, Preparacrista orientation: (0) angled buccally; (1) straight; (2) crest absent.

160. D, M^1, Paracone and metacone relative sizes: (0) paracone larger than metacone or metacone absent; (1) cusps are subequal; (2) metacone larger than paracone.

161. D, M^1, Paracone and metacone bases relationship (0) M^1 paracone and metacone clearly separated at their bases; (1) M^1 paracone and metacone connate (no separation at the bases of the cusps).

162. D, M^1, Metastylar region buccal projection: (0) greater than parastylar region; (1) less than or equal to parastylar region.

163. D, M^1, Metastyle presence: (0) absent; (1) present.

164. D, M^1, Post- and metacingula continuity: (0) not continuous; (1) continuous.

165. D, M^1, Conules presence: (0) both conules present; (1) metaconule absent; (2) both weak or absent; (3) paraconule absent.

166. D, M^1, Conules position: (0) central or closer to protocone than to paracone and metacone; (1) appressed to paracone and metacone.

167. D, M^1, Protocone size relative to the buccal half of the tooth: (0) large; (1) small.

168. D, M^1, Protocone position: (0) skewed mesiobuccally; (1) central on the tooth.

169. D, M^1, Protoloph presence: (0) absent; (1) present.

170. D, M^1, Hypocone presence: (0) absent; (1) present (true hypocone, coming off the cingulum); (2) present (pseudohyopcone, budding off the postprotocingulum).

171. D, M^1 or M^2, Postprotocingulum presence: (0) absent; (1) weak; (2) pronounced.

172. D, M^2, Ectoflexus depth: (0) deep with the stylar shelf wide at the corners and almost disappearing in the middle; (1) shallow.

173. D, M^2, Hypocone size: (0) small, distinctly smaller than the protocone; (1) large, similar in size to the protocone; (2) hypocone absent.

174. D, M^3, Presence: (0) present; (1) absent.

175. D, M^3, Relative size based on (ln(buccal L x W)M^3) (ln(buccal LxW)M_1): (0) more than 0.9; (1) less than or equal to 0.9.

176. D, M^3, Prominent parastylar lobe presence: (0) not prominent or absent; (1) prominent.

177. D, M^3, Metacone presence: (0) metacone present as a well-developed cusp; (1) metacone absent.

178. D, M^3, Hypocone size: (0) very small or absent; (1) large.

179. D, Upper Molar, Stylar shelf morphology: (0) broad; (1) narrow (buccal cingulum only) or absent.

180. D, Upper Molar, Mesostyles presence (0) absent; (1) one or more present.

181. D, Upper Molar, Centrocrista morphology: (0) moderate; (1) strong and straight; (2) absent or very weak; (3) strong and V-shaped.

182. D, Anteriormost Lower Incisor, Continuous growth presence: (0) absent; (1) present.

183. D, Anteriormost Lower Incisor, Enamel restricted to an anterior band: (0) not restricted; (1) restricted.

184. D, Anteriormost Lower Incisor, Root extent relative to M_3: (0) does not extend below M_3; (1) extends below M_3.

185. D, I_1, Size: (0) much larger than other incisors (or premolars if I_{2-3} are lost); (1) comparable to other incisors (or premolars if I_{2-3} are lost); (2) very reduced; (3) tooth absent

186. D, I_1, Form: (0) simple, not laterally compressed; (1) laterally compressed with no broad, flattened surface; (2) as 1, with flattened dorsal surface; (3) as 2, but rotated medially.

187. D, I_1, Orientation: (0) essentially vertical (between vertical and 45 degrees); (1) procumbent-horizontal (greater than 45 degrees).

188. D, I_1, Margoconid presence: (0) absent; (1) present.

189. D, I_2, Presence: (0) present, large and/or larger than i1; (1) present, small; (2) absent.

190. D, I_3, Presence: (0) present; (1) absent.

191. D, C_1, Lower canine root number: (0) one; (1) two; (2) tooth absent.

192. D, C_1, Lower canine relative size: (0) larger than adjacent teeth; (1) smaller than adjacent teeth; (2) tooth absent.

193. D, P_1, Presence: (0) present; (1) absent.

194. D, P_2, Alveoli number: (0) two; (1) one; (2) tooth absent.

195. D, P_2, Crown, Large, procumbent, with a hatchet-like slicing anterior projection: (0) absent; (1) present.

196. D, P_3, Root number: (0) two; (1) one; (2) tooth absent.

197. D, P_3, Paraconid (anterior basal cusp) presence: (0) present; (1) absent.

198. D, P_4, Number of roots: (0) one; (1) two roots fused with apical division; (2) two.

199. D, P_4, Mesiodistal length relative to M_1: (0) P_4 somewhat shorter than M_1; (1) P_4 and M_1 subequal in length; (2) P_4 much longer than M_1; (3) M_1 much longer than P_4.

200. D, P_4, Paraconid presence: (0) paraconid distinct, cuspate; (1) cusp indistinct but paracristid present, not markedly elongate; (2) paraconid and paracristid absent or weak; (3) paracristid elongate with or without a distinct cusp.

201. D, P_4, Metaconid presence: (0) absent; (1) present.

202. D, P_4, Cristid obliqua position: (0) joins postvallid near midline of tooth or more lingually; (1) joins postvallid near buccal margin of trigonid; (2) cristid obliqua absent.

203. D, P_4, Hypoflexid morphology: (0) distinct, deep; (1) not distinct, shallow.

204. D, P_4, Talonid morphology: (0) basined; (1) not basined.

205. D, P_4, Talonid cusp number: (0) three well defined; (1) two well defined; (2) one solo distinct cusp; (3) all poorly defined.

206. D, M_1, Crown height (M_1 trigonid height over tooth length): (0) high crowned (index value more than 0.79); (1) moderate (index value 0.60-0.78); (2) low crowned (index value less than 0.6).

207. D, M_1, Trigonid degree of mesiodistal compression: (0) strongly compressed mesiodistally; (1) longer, with the paraconid positioned more mesially relative to the metaconid.

208. D, M_1, Trigonid height: (0) taller than the talonid but less than two times the height of the talonid; (1) of a similar height to talonid; (2) trigonid two times taller than the talonid or more.

209. D, M_1, Trigonid basal: (0) not swollen at the base; (1) swollen basally.

210. D, M_1, Mesiobuccal projection presence: (0) absent; (1) present.

211. D, M_1, Paraconid distinctiveness from paracristid: (0) indistinct from paracristid; (1) distinct from paracristid.

212. D, M_1, Paraconid size: (0) large; (1) small, markedly smaller than metaconid; (2) paraconid absent.

213. D, M_1, Protoconid and metaconid relative height: (0) protoconid higher; (1) subequal; (2) metaconid higher.

214. D, M_1, Metaconid position relative to protoconid: (0) metaconid and protoconid in line or metaconid in front of protoconid; (1) metaconid positioned well behind the level of the protoconid.

215. D, M_1, Stepped postvallid presence: (0) absent; (1) present.

216. D, M_1, Talonid basin form: (0) talonid with well defined basin surrounded by ridges continuous with or comprised of the entoconid, hypoconid, and or hypoconulid; (1) talonid basin reduced or absent.

217. D, M_1, Talonid width near cusp apices: (0) somewhat narrower than trigonid; (1) wider than trigonid; (2) much narrower than trigonid (the distance between the lingual margin and the point at which the cristid obliqua contacts the postvallid is less than half the width of the trigonid).

218. D, M_1, Talonid cusps relative height: (0) hypoconid taller than entoconid; (1) entoconid taller than hypoconid.

219. D, M_1, Entoconid notch presence (between the entoconid and hypoconulid): (0) absent; (1) present.

220. D, M_1, Hypoconulid notch presence (between the hypoconulid and hypoconid): (0) absent-weak; (1) present.

221. D, M_1, Hypoconulid and entoconid relative positions (see ref. 24: fig. 3.18): (0) unpaired; (1) paired (hypoconulid lingual of the central axis of the tooth but not directly appressed

to the entoconid); (2) twinned (hypoconulid appressed to the entoconid in the distolingual corner of the tooth).

222. D, M_1, Hypoconulid position relative to the central axis of the tooth: (0) hypoconulid centrally placed or lingual of the central axis of the tooth; (1) hypoconulid buccal of central axis; (2) cusp absent.

223. D, M_2, Mesiobuccal projection presence: (0) absen; (1) present.

224. D, M_2, Paraconid distinctiveness compared to M_1: (0) comparably distinct to the paraconid on m1; (1) less distinct than the paraconid on M_1.

225. D, M_3, Presence: (0) present; (1) absent.

226. D, M_3, Length relative to M_2: (0) M_3 less than or equal to M_2; (1) M_3 greater than M_2.

227. D, M_3, Talonid width: (0) much narrower than trigonid; (1) similar in breadth to trigonid or wider; (2) talonid absent.

228. D, M_3, Hypoconulid size: (0) similar to that cusp on $M_{1\text{-}2}$; (1) larger than on $M_{1\text{-}2}$ but not developed into a lobe; (2) developed into a lobe.

229. D, Lower Molar, Relative sizes: (0) lower molars get progressively smaller distally from $M_{1\text{-}3}$; (1) lower molars get progressively larger from $M_{1\text{-}3}$; (2) all lower molars similar in size; (3) M_2 is the smallest lower molar; (4) M_2 is the largest lower molar.

230. D, Lower Molar, Lingual curvature ("sweeps smoothly from the paraconid to the rear of the talonid"; McKenna, 1963: p. 17): (0) absent; (1) present.

231. D, Lower Molar, Trigonid length along the tooth row: (0) trigonids become less mesiodistally compressed from $M_{1\text{-}3}$; (1) no change; (2) trigonids become more mesiodistally compressed from $M_{1\text{-}3}$.

232. D, Lower Molar, Trigonid mesial inflection presence (in mesially oriented trigonids the postvallid is at a greater than ninety degree angle to the floor of the talonid basin; see ref. 24: fig. 3.13): (0) absent; (1) weak; (2) pronounced.

233. D, Lower Molar, Curving paracristids presence: (0) absent; (1) present.

234. D, Lower Molar, Protoconid-metaconid notch morphology: (0) strong and sharp; (1) more rounded; (2) fold of enamel bridges notch.

235. D, Lower Molar, Buccal cingulid(s) presence: (0) anterobuccal "precingulid" only; (1) separate anterior and posterior cingulids; (2) continuous buccal cingulid; (3) absent (no buccal cingulid or "precingulid").

236. D, Lower Molar, Hypoflexid distinctiveness: (0) distinct, invaginated; (1) not distinct

237. D, Molar, Enamel roughness: (0) smooth; (1) crenulated.

238. D, Molar, Cusp acuteness: (0) relative acute; (1) blunter (more bunodont).

239. D, Diastema, Large upper diastema presence: (0) diastema absent or shorter than the molar toothrow; (1) diastema longer than the molar toothrow.

240. D, Diastema, Diastema between the lower incisors and the cheek teeth presence: (0) absent; (1) present.

Table S2. Taxon-character matrix formatted facilitating importation to a parsimony computer program, such as TNT [7].

```
#NEXUS

BEGIN TAXA;
      DIMENSIONS NTAX=30;
      TAXLABELS
            Ukhaatherium Leptictidae Erinaceus Echinosorex Hemiechinus
Ottoryctes Pararyctes Paleoryctes Uintacyon '''Vulpavus profectus'''
Solenodon '''Sorex cinereus sorex sp.''' '''Suncus murinus''' Rhombomylus
'''Ptilocercus lowii''' '''Tupaia glis''' Genetta '''Cynocephalus
volans''' Gypsonictops Purgatorius Carpolestidae Plesiadapidae
Paromomyidae Microsyopidae Micromomyidae '''Altanius orlovi''' Adapidae
Omomyidae Torrejonia_wilsoni Plesiolestes_nacimienti
      ;

END;


BEGIN CHARACTERS;
      DIMENSIONS  NCHAR=240;
      FORMAT DATATYPE = STANDARD GAP = - MISSING = ? SYMBOLS = "  0 1 2 3
4";
      MATRIX
      Ukhaatherium
10100010000000000000??00?1001100100000000020020000?1001?000101000?000000110
?0210?11100?101??021?00110?0000?000000000001000200210010102000100000011000
00000102?0001110001100002001000000001010100000000201000120020011000000001000
0000002000000000000
      Leptictidae
?01100010000100000?1??210?2110111001100110000101020000000001?10000?10122100
1100020101000101001110000101100?0220011 0?0?011002002101021020002010000030 0
0000010000100010011000000011000000000100010200000120(0
1)1000000000110000 0?000000000030000000000
      Erinaceus
200101102000210011000000100101000100102?002001211010011?0001000000020112100
011002110010110001210001110100 0?001000110000111010200101000210001111111010
11011000111100113000110010111000000000010110012?112001211311110101000 11???2
000020002000200000
      Echinosorex
210101112000?10012000011002110011001??010200021100001100001000000001001210 0
001002010010110001100001 10?1000?00100011000010101002100101021001111001101 0
1101110011110011300011011010011000000010000001001200001032110010100011???2
000000002000200000
      Hemiechinus
210110102000210000???0110100000100112???????????????????????0???01020102100
012012(0
1)10010111001210001110100 0?001000110000111020020001010210011111011010110 11
1001111001 12?0011011011101000000001011001 2?11200001032110010200011000000002
0002000300000
```

Ottoryctes
??0?????????????????021(0
1)00??2?0?010100011?001101?1110111100210011 00??010112102101100020001011000
1000000000000000111101011000020010000000002000000011000210000120020011000020
00000001002110000000000
Pararyctes
?????????????????????????1?????????110????????????????????????????????02?000
??1?020?0?0??11??011?0011101?00?0210?10??????011101200010002000201100 11000
000001000000101 02?110100000100000000 3???010011011200000020020011000020000 0
0001011110000000000
Paleoryctes
???????10??0??0??0??
????0?????0??01???12?????10????????????1???????01200200110002000001110?1000
10000102?000111?0011000020010000 0?0020000000110002100001200200010000200000
0000002110000000000
Uintacyon
??0??1??
????????????????????????????????????0?0?????0?00020101101000200120110011010
100000000000001101010000120???000000????100000000200011110120010000020???2
0000000012000000000
'''Vulpavus profectus'''
?010000100000?0?0010??21??100?00001000002000000010200 00?1?00011?00??0011100
?11000??0?01000?1112000?00?0002?0010?0?000001000200011010002001001100 11010
1100000100000101200001 01?0110000000010001000000001200011121100010000000???2
0000100020000000000
Solenodon
1011011101100000120000011010001010111101120101010000000000010000010100120 10
000100010100(0 1)00001020?011100000?001001(0 1
2)100001 0100?101100000201020100011010110110 02?000?11?2?0101001001?000?0002
011100000002?2301211301200110001 2????2000100201000000000
'''Sorex cinereus sorex
sp.'''20111000010000 00?2????111010101010010?00020102?(0
1)000011000010100000200000100001001000 0?000000020001110000 0?0011000111001 0
0110200100000210011110011010100101100102001100000101001110013 00?0111210112
?2?03112113010001000001110200000000101100200000
'''Suncus murinus'''
20111100011 0?100??110001200010101011 02?1020112110000110000101 00?0010002010
0001001100010000010200011100000?001100?11100210110200100000200010110011010
0001011000020011 2?0001010011100130000110210112?2?0310211301000100000111020
0000000101000200000
Rhombomylus
2000011100 0?100?00?0?0?1??201001100010?11100010011 0?00??00??1?0?00??122111
00200201000??01?0000?1021100122?0010?0120?1101210?0010(0
1)1010211010110 1(0
1)1010001001 02?12001112?011101100001102?113???212212?00200100000000022000
0111010001011011110 00111
'''Ptilocercus lowii'''
1010000110110001001 0??10110?000000000010000020010201000101001011021111221 0
112101010001001100101010110111111211001100002111200010000102100 10?11010010
111(0 1)110101120011 2?000101001100000000 1010000011011230 1(0
2)0130100010000001102000000000101 0200000
'''Tupaia glis'''
10010001000000000001000210221001100000010000022010201000001001000 02?0022210
112002000000000110011101010?11111021000110000210120021000 0(0

1)02000101110100001110111 2?00201102?0001000011000130001010000011 0012301(0
2)013010001000001110200000000001010300000
Genetta
2010001100001000011000110210100100100001120001110200000000000001000030122100
101(0 1)0100010(0
1)0101102000010 0?1002?020010100000100020021001010200120110011010100010 00?0
01010?2?00000021?1??000000100010010000020012113112001000 0000???2011???0020
00000000
'''Cynocephalus volans'''
1010001000100111001010001100000000000000000100200002100110010010211031121110
1120021100000001000 2?10200?1022?0101101000002011 0?0211100002100201111 00310
00000112?102010000010001200000000 30001?10001010?012101100001100100100101120
000010002000301000
Gypsonictops
??
??011011010200020110001310
0001?100000?0??001100000200100000??????????0?000012101000(0
1)000101110001???000000000201010000000
Purgatorius
???001002001021000????????????????????
???1120???????021010010200010110000000
001001000000000000(0 1)00(0 1)000100000?0?0?1?10000000120000000101000(0
1)10000(0 1)0000000010110(0 1)1100000?0
Carpolestidae
?010000110110 00?000021011110011 0?0000000010020010210001110011020???0122100
002112100?0??01?0021?0??111?100?1??0?0?113002000100100101102100211 10101111
0110010001100100010101210000001000000211100011020220120120101010011011 0000
000112302110000000
Plesiadapidae
?0100001001000000000000111100010000000000000000200102(0
1)00001000?0010001012210101 21?210??0??01?0021001?1111102?1?00?011130011012
1011010110111 00011(0 1)1001100110010(0
1)11100101010100210000001020000211110111001232 0201311(0 1)1010(0
1)11011000000011210211 1000100
Paromomyidae
?1100001101000010000111112100100000000000000000200102(0
1)00000000?102?01?0120100?0211211??0??1111121?110110 1112?122000111 30011012
?001?000002100(0 2)011(0 1)01001001100100010000011100002100100010 00000(1
2)10110(0 1)100002010000111101111000110000010112102212201000
Microsyopidae
????????????10??????????????????????????????????????1?????????????????110110
1001101000000101112(0
1)11??0101000?0?1000111100211120100100000100?1111001000001110100011 0011001
0001100011001000000310210111001201100011100 1110?001001100000013011 10000000
Micromomyidae
00100001001000010100111011 0002?01000000000100200102100000 0?00102???101??100
?02????????????1???21?1101???002?1??0?011120021112002110000020001011 0000010
000001000000000000100000000010010000000110210110001223 0000(1 3)(0
1)10010100001(0 1)01101001013 00110(0 1)00000
'''Altanius orlovi'''
??
??002?0210000102000?0110010001
11?0010001110100000101112010001 00??01?0?1100000012001000101010100110110000
000112102010200000

```
      Adapidae
?010000110110000000002121122?0201000000000000200102110111100110000?10212200
0111021000000001100211010111111111010?0100000(1
2)100200010010102100101101100001110010(0 1)0110010100000(0
1)11201000100000100011000000120(0 1)1(0 1)(0 1)(0
1)311010100110100001000101102010(0 2)00(0 1)00
      Omomyidae
?01100011?11000???????????11121110000000001002200021101??1??1????????2?220?
0121021?000?011??021101?1111111?10?0?0?(0 1)(0 1)100(1
2)1001001110001021000011(0 1)(0 1)1000011100100011001000(0 1)010(0
1)1120(0 1)0001000001(0 1)00110001001200100011101010 01(0 1)010000000010(1
2)302(0 1)10200000
      Torrejonia_wilsoni
?01???011?11000??0????1?1???110??0?000000000?00?021000????????2???????????
???????????????????????????????????0?011110????11?1????????210000110100(2
3)10000001000111010101000021000000100000021011001100121(0 1)(0
1)100111010111000100000010112102111 2011?0
      Plesiolestes_nacimienti
?????????????????????????????????????????????????????????????????????????0?
?02???????????????????????????????????0????????01100(0 2)(0 1)1100(0
1)0210000110000(0 2)101(0 1)100100000(0 1)000(0 1)0100002120(0
1)000100??00?1?00001100120?(0 1)00010100011100010010001011 2202111(0
1)00000

;

END;
```
